# Two 18‐norspirostane steroidal saponins as novel mitophagy enhancers improve Alzheimer's disease

**DOI:** 10.1002/ctm2.1390

**Published:** 2023-09-01

**Authors:** Wen‐Qiao Qiu, Lu Yu, Chang‐Long He, Jian‐Ming Wu, Betty Yuen‐Kwan Law, Chong‐Lin Yu, Da‐Lian Qin, Xiao‐Gang Zhou, An‐Guo Wu

**Affiliations:** ^1^ Sichuan Key Medical Laboratory of New Drug Discovery and Druggability Evaluation Luzhou Key Laboratory of Activity Screening and Druggability Evaluation for Chinese Materia Medica School of Pharmacy Education Ministry Key Laboratory of Medical Electrophysiology Southwest Medical University Luzhou China; ^2^ Department of Neurosurgery Sichuan Provincial People's Hospital University of Electronic Science and Technology of China Chengdu China; ^3^ State Key Laboratory of Quality Research in Chinese Medicine Macau University of Science and Technology Taipa China

Dear Editor,

The hallmark of Alzheimer's disease (AD) is the progressive accumulation of misfolded proteins, specifically beta‐amyloid (Aβ) and Tau, leading to neurotoxicity and impaired neuronal function.^1^ Impaired mitophagy in AD contributes to protein buildup and subsequent neuronal damage.^2^ Mitophagy enhancers are investigated as potential therapeutic interventions for AD.^3^, ^4^ Deoxytrillenoside CA (DTCA) and epitrillenoside CA (ETCA), two newly discovered compounds derived from *Trillium tschonoskii* Maxim. (TTM) (Figure 1A), have shown antioxidative properties by inducing autophagy.^5^ Nevertheless, their impact on mitophagy induction and clearance of AD‐related proteins remains unexplored.

**FIGURE 1:**
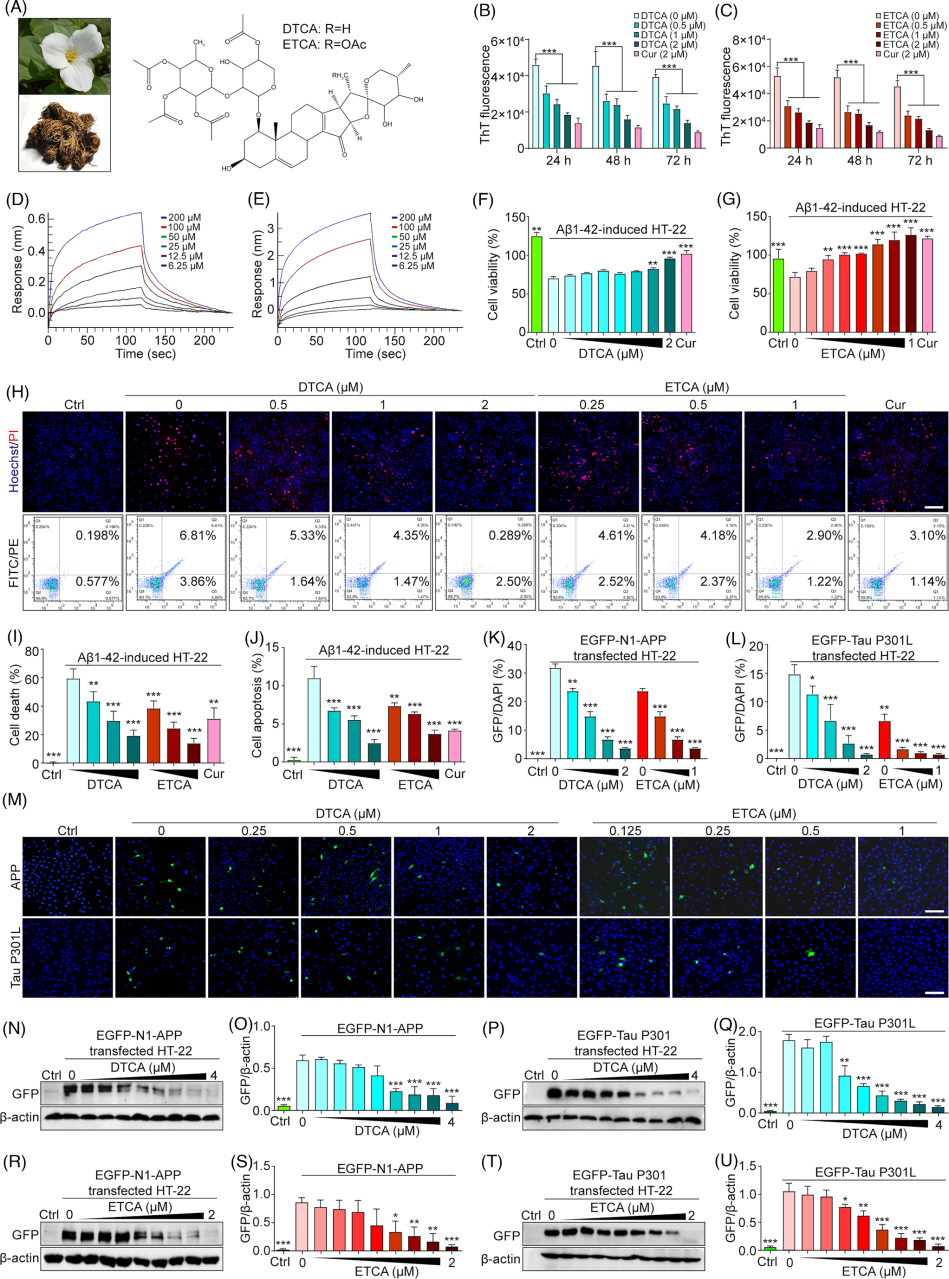
Inhibition of Aβ1‐42 fibrillization, cytotoxicity and AD‐related protein expression by deoxytrillenoside CA and epitrillenoside CA (DTCA&ETCA). (A) Aerial part and rhizome of TTM and chemical structures of DTCA&ETCA. (B and C) Bar charts showing the fluorescence intensity of solutions containing ThT reagent and Aβ1‐42 with or without DTCA, ETCA, and Curcumin (Cur) at specified concentrations; error bars, ***p < .001, *n* = 3. (D and E) Kinetic analysis of DTCA and ETCA binding affinity to Aβ1‐42 using BLI assay. The response (nm) represents the optical thickness of the sensor layer, reflecting the spectral shift (Δλ) during the interaction of DTCA (6.25–200 μM) or ETCA (6.25–200 μM) with Aβ1‐42. (J and K) Bar charts indicating the cell viability of HT‐22 cells treated Aβ1‐42, with or without DTCA and ETCA at specified concentrations, using Cur (10 μM) as a positive control; error bars, standard deviation (S.D.), *p < .05; **p < .01; ***p < .001, *n* = 3. (H) Representative images of Hoechst/PI staining and flow cytometry images of Aβ1‐42‐treated HT‐22 cells with or without DTCA and ETCA at specified concentrations, using Cur (10 μM) as a positive control. (I and J) Bar charts indicating the cell death and cell apoptosis of HT‐22 cells; error bars, S.D., **p < .01; ***p < .001, *n* = 3. (K and L) Bar charts showing GFP/DAPI ratios in HT‐22 and PC‐12 cells; error bars, S.D., *p < .05; **p < .01; ***p < .001, *n* = 3. (M) Representative images of merged GFP and DAPI in HT‐22 cells overexpressing EGFP‐N1‐APP or EGFP‐Tau P301L and stained with DAPI, treated with DTCA and ETCA at specified concentrations. Magnification: 20× , scale bar: 100 μm. (N, R) Representative Western blot images of GFP and β‐actin in HT‐22 cells overexpressing EGFP‐N1‐APP and treated with DTCA and ETCA at specified concentrations. (O, S) The relative protein expression of APP is indicated by the ratio of GFP‐tagged APP to β‐actin using a GFP antibody, represented as GFP/β‐actin. Bar charts indicating GFP/β‐actin ratios in HT‐22 cells; error bars, S.D., *p < .05; **p < .01; ***p < .001, *n* = 3. The original Western blot images are presented in Figure S19, where the protein molecular weight markers were labelled. (P, T) Representative Western blot images of GFP and β‐actin in HT‐22 cells overexpressing EGFP‐Tau P301L and treated with DTCA and ETCA at specified concentrations. (Q, U) The relative protein expression of Tau P301L is indicated by the ratio of GFP‐tagged Tau P301L to β‐actin using a GFP antibody, represented as GFP/β‐actin. Bar charts displaying GFP/β‐actin ratios in HT‐22 cells; error bars, S.D., *p < .05; **p < .01; ***p < .001, *n* = 3. The original Western blot images are presented in Figure S19, where the protein molecular weight markers were labelled.

In this study, our primary focus was to investigate the autophagic degradation effects of DTCA&ETCA on AD‐related proteins. To begin, the Aβ fibrillization was measured using the Thioflavin T (ThT) reagent. The results showed that DTCA&ETC, administrated at relatively safe concentrations (Figure S1), exhibited a dose‐dependent reduction in ThT fluorescence, indicating the inhibition of Aβ1‐42 (Figure 1B,C) and Aβ25‐35 fibrilization (Figure S2A,B). The biolayer interferometry (BLI) analysis displayed that DTCA&ETCA directly bound to Aβ1‐42 (Figure 1D,E). Subsequently, the effect of DTCA&ETCA on Aβ’s cytotoxicity was examined, revealing an increase in cell viability and a decrease in cell death and apoptosis of a immortalized mouse hippocampal neuronal cell line (HT‐22) or a rat adrenal pheochromocytoma cell line (PC‐12) cells (Figure 1F‐J, Figure S2C‐I, S3). To investigate how DTCA&ETCA influence the removal of AD‐associated proteins, the green fluorescent protein (GFP) fluorescence intensity and protein expression in neuronal cell lines transfected with enhanced green fluorescent protein fused amyloid precursor protein (EGFP‐fused APP), Tau, or Tau P301L plasmid were determined. Figure 1K‐U and Figure S4‐6 clearly demonstrated that DTCA&ETCA significantly hinder the GFP intensity and protein expression, while not affecting transfection efficacy. Furthermore, the 3‐(4,5‐Dimethylthiazol‐2‐yl)‐2,5‐diphenyl‐2H‐tetrazolium bromide (MTT) results indicated that DTCA&ETCA enhanced the viability of transfected neuronal cells (Figure S7). Moreover, the flow cytometry analysis revealed that DTCA&ETCA not only decreased GFP intensity but also reduced the proportion of propidium iodide (PI)‐positive cells (Figure S8). Collectively, DTCA&ETCA effectively inhibits Aβ production and facilitates the clearance of AD‐related proteins.

Autophagy activity was assessed by examining the conversion of microtubule‐associated protein 1A/1B‐light chain 3 (LC3), the number of LC3 puncta, and autophagy flux.^5^, ^6^ DTCA&ETCA increased LC3 conversion and puncta in a dose‐ and time‐dependent fashion (Figure 2A‐C, Figure S9). Furthermore, DTCA&ETCA induced autophagy flux as indicated by the elevated red fluorescent protein‐LC3 (RFP‐LC3)/GFP‐LC3 ratio (Figure 2D,E). Notably, autophagy flux induced by DTCA&ETCA was impeded by 3‐methyladenine (3‐MA) and bafilomycin A1 (Baf), demonstrated by reduced LC3 conversion and puncta during autophagy initiation and increased LC3 conversion and puncta during autophagosome‐lysosome fusion (Figure 2F, Figure S10). The transmission electron microscopy images revealed increased engulfment of mitochondria (mt) by autophagosomes (av) in HT‐22 cells treated with DTCA, ETCA, or urolithin A (UA, a positive mitophagy activator), suggesting the potential induction of mitophagy by DTCA&ETCA (Figure 2G). Mechanistic investigations indicated that DTCA&ETCA inhibited mTOR and activated the AMPK/ULK1 and PINK1/Parkin pathways (Figure 2H, Figure S11). Cotreatment with compound C (CC) and SBI0206965 (SBI) attenuated LC3 conversion and GFP‐LC3 puncta induced by DTCA&ETCA (Figure S12). Moreover, SBI significantly reversed the elevated colocalization of GFP‐LC3 with mitochondria and the reduced GFP/RFP ratio in DTCA&ETCA‐treated HT‐22 cells transfected with Mito‐QC, a construct expressing a mCherry‐GFP tag attached to the outer mitochodrial membrane FIS1 (residues 101‐152) (Figure 2I‐L). To investigate whether DTCA&ETCA degrade AD‐associated proteins via mitophagy, 3‐MA and Baf were employed. Figure 2M‐O and Figure S13‐14 demonstrated that 3‐MA and Baf abolished the reduction in GFP expression induced by DTCA and ETCA in HT‐22 cells transfected with EGFP‐fused APP, Tau or Tau P301L plasmid. Additionally, MEF cells with or without the Atg7 gene were utilized to explore how Atg7 regulates the clearance of AD‐associated proteins by DTCA&ETCA. Figure S15 showed that DTCA&ETCA activated autophagy and significantly reduced GFP intensity in wild‐type MEFs, while this effect is absent in Atg7‐deficient MEFs. Furthermore, DTCA&ETCA inhibited cell apoptosis in EGFP‐N1‐APP‐ or EGFP‐Tau‐P301L‐transfected HT‐22 cells (Figure S16). Overall, DTCA&ETCA promote the autophagic degradation of AD‐related proteins, leading to the alleviation of neuronal apoptosis.

**FIGURE 2:**
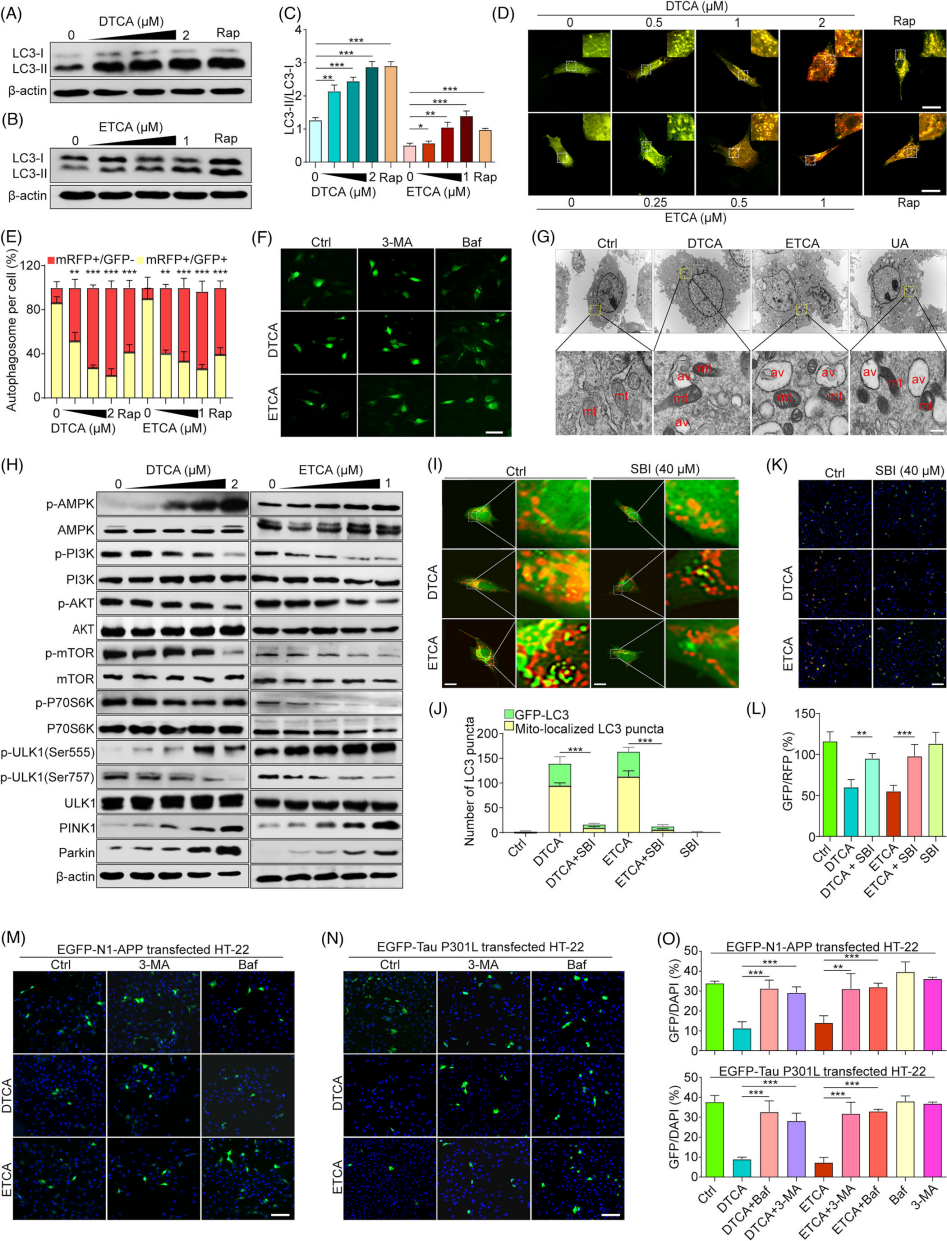
Activation of mitophagy by deoxytrillenoside CA and epitrillenoside CA (DTCA&ETCA) through mTOR, AMPK/ULK1 and PINK1/Parkin signaling pathways, and promotion of autophagic degradation of AD‐related proteins. (A and B) Western blot images of LC3 and β‐actin in HT‐22 cells treated with DTCA, ETCA and Rapamycin (Rap) at specified concentrations for 24 h. The original Western blot images are presented in Figure S21, where the protein molecular weight markers were labelled. (C) Bar chart indicating LC3‐II/LC3‐I ratios; error bars, S.D., *p < .05; **p < .01; ***p < .001, *n* = 3. (D) Merged images of GFP‐LC3 and RFP‐LC3 in tf‐LC3‐overexpressing HT‐22 cells treated with DTCA, ETCA and Rap at specified concentrations. Magnification: 63× , zoom in, scale bar: 5 μm. (E) Bar chart indicating the percentage of autophagosome per cell in tf‐LC3‐overexpressing HT‐22 cells treated with DTCA, ETCA and Rap at specified concentrations; error bars, S.D., **p < .01; ***p < .001, *n* = 3. (F) Representative images of GFP‐LC3 puncta in HT‐22 cells treated with DTCA (.5 μM) and ETCA (.25 μM) with or without 3‐MA (5 mM) and Baf (5 nM). Magnification: 40× , scale bar: 25 μm. (G) Electron micrographs of HT‐22 cells treated with or without DTCA (.25 μM), ETCA (.5 μM), and UA (50 μM) for 24 h, showing ultrastructural details. Top: Magnification: 10,000 × , scale bar: 2 μm; Bottom: Magnification: 250 000× , scale bar: 500 nm. Autophagic vacuoles (av) and mitochondria (mt) are indicated. (H) Western blotting of phosphorylated AMPK (p‐AMPK), AMPK, p‐PI3K, PI3K, p‐Akt, Akt, p‐P70S6K, P70S6K, p‐mTOR, mTOR, p‐ULK1(Ser555), p‐ULK1(Ser757), ULK1, PINK1, Parkin and β‐actin in HT‐22 cells exposed to DTCA and ETCA at specified concentrations for 24 h. The original Western blot images are presented in Figure S22, where the protein molecular weight markers were labelled. (I) Merged images displaying GFP‐LC3 puncta and mitochondria in GFP‐LC3‐expressing HT‐22 cells, alongside Mito‐Tracker Red CMXRos‐stained mitochondria. The cells were treated with DTCA and ETCA with or without SBI for 24 h. The image on the right, featuring enhanced detail, corresponds to the zoomed‐in region delineated by the white dashed line. Magnification: 40×, scale bar: 10 μm. (J) Quantification of GFP‐LC3 puncta (green) and Mito‐localized LC3 puncta (yellow); bars, S.D., ***p < .001, *n* = 3. (K) Merged images of GFP and RFP in Mito‐QC‐expressing HT‐22 cells exposed to DTCA and ETCA with or without SBI for 24 h; magnification: 10× , scale bar: 100 μm. (L) Quantification of GFP/RFP in Mito‐QC‐expressing HT‐22 cells; bars, S.D., **p < .01; ***p < .001, *n* = 3. (M and N) Representative images of EGFP‐N1‐APP‐ or EGFP‐Tau P301L‐expressing HT‐22 cells treated with DTCA (0.5 μM) and ETCA (0.25 μM) with or without 3‐MA (5 mM) and Baf (5 nM) for 24 h. Magnification: 20× , scale bar: 100 μm. (O) Bar charts showing GFP/DAPI ratios in EGFP‐N1‐APP‐ or EGFP‐Tau P301L‐expressing HT‐22 cells; error bars, S.D., **p < .01; ***p < .001, *n* = 3.

To investigate the potential of DTCA&ETCA in stimulating mitophagy in vivo, BC12921, DA2123 and IR1631 strains were utilized.^7^ Figure 3A‐D demonstrated that DTCA&ETCA resulted in an elevation of GFP‐LGG‐1 foci in DA2123 worms and a decline in p62‐GFP protein expression in BC12921 worms. Additionally, DTCA&ETCA significantly decreased the GFP/DsRed ratio in IR1631 worms, implicating the involvement of *unc‐51* and *pdr‐1* genes (Figure 3E,F). These findings collectively suggest that DTCA&ETCA induce mitophagy in *Caenorhabditis elegans*. Subsequently, CL2331, CL4176 and BR5270 strains were employed to investigate the anti‐AD effects of DTCA&ETCA.^8^ Figure 3G,H demonstrated that DTCAT&ETCA significantly reduced Aβ aggregations in the anterior region of CL2331worms. Moreover, in CL4176 worms, DTCA&ETCA exhibited a delay in Aβ‐induced paralysis (Figure 3G,I), while in BR5270 worms, it alleviated the food‐searching deficit (Figure 3J). When we fed CL4176 worms with RNAi *unc‐51*, RNAi *Pdr‐1*, RNAi *bec‐1* and RNAi *vps‐34* bacteria, the paralysis got worse and the delay effect of DTCA&ETCA on paralysis disappeared (Figure 3K,L). These results strongly support the notion that DTCA&ETCA exert potent anti‐AD effects by inducing mitophagy in *C. elegans*. Subsequently, we studied the impact of ETCA on AD using APP/PS1 mice. The result showed that the cognition examined by Morris Water Maze was greatly alleviated by ETCA (Figure 4A‐D). Furthermore, ETCA significantly reduced Aβ and p‐Tau levels, the Bax/Bcl‐2 ratio, GFAP, and Iba‐1 contents, while increasing NeuN‐positive cells in the brain tissue (Figure 4E‐I, Figure S17A,B). Mechanistically, ETCA significantly increased the phosphorylation (p) of AMPK, p‐ULK1(Ser555), LC3‐II, Parkin and PINK1, while down‐regulating p‐mTOR, p‐ULK (Ser757) and p‐70S6K in the brain tissue (Figure 4J‐L, Figure S17C,D). Collectively, ETCA improves cognition and pathology of APP/PS1 mice potentially via mitophagy induction.

**FIGURE 3:**
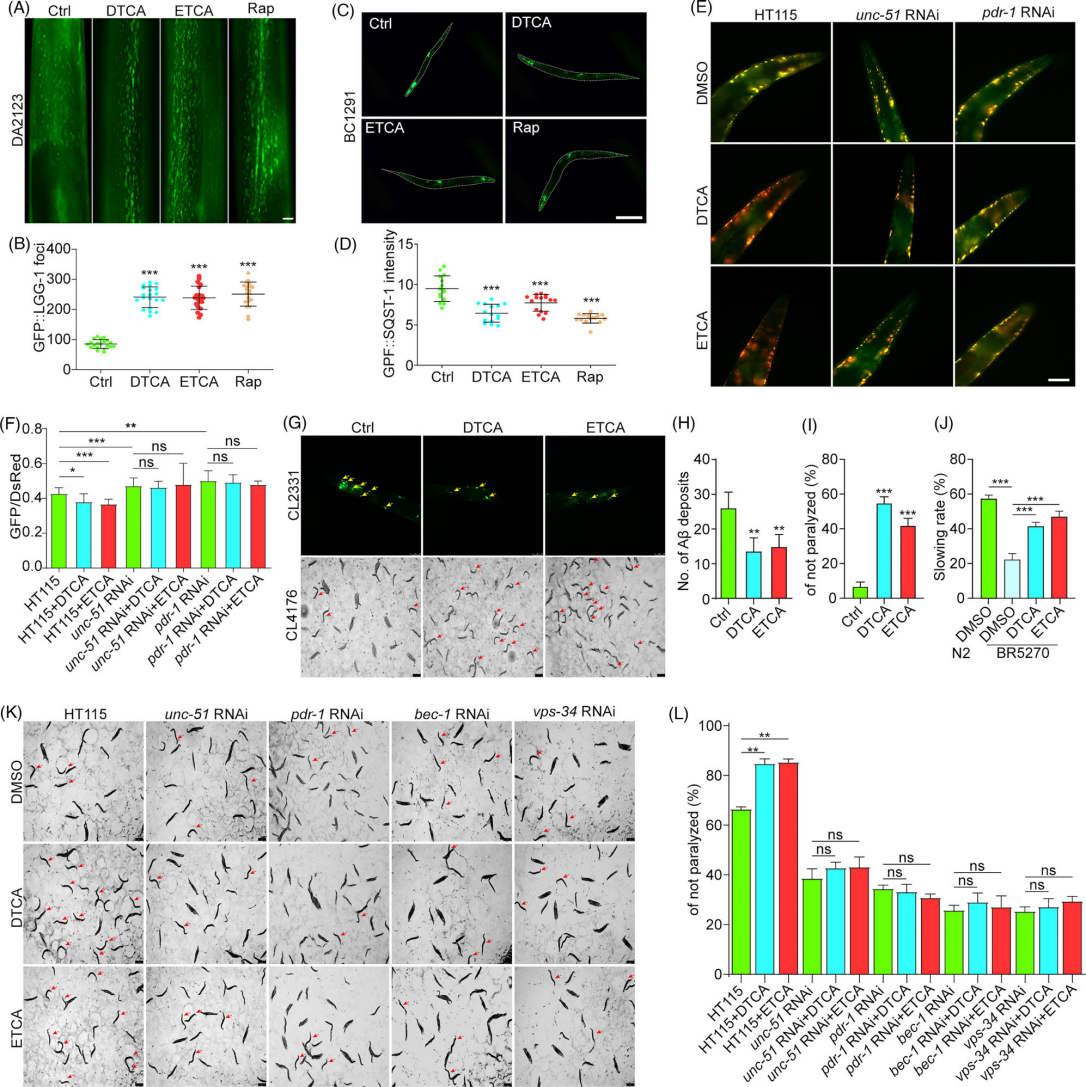
Deoxytrillenoside CA and epitrillenoside CA (DTCA&ETCA) alleviate pathology and behaviour deficits in *Caenorhabditis elegans* models of AD through mitophagy induction. (A) Representative images of GFP::LGG‐1 foci in DA2123 worms treated with or without DTCA, ETCA and Rap for 48 h. Magnification: 40× , scale bar: 25 μm. (B) Bar chart showing the number of GFP::LGG‐1 foci; error bars, S.D., ***p < .001, *n* = 20. (C) Representative images of SQST1‐GFP expression in BC12921 worms treated with or without DTCA, ETCA and Rap for 48 h. Magnification: 10× , scale bar: 100 μm. (D) Bar chart displaying relative expression of SQST‐1‐GFP; error bars, S.D., ***p < .001, *n* = 20. (E) Representative images of merged pH‐sensitive GFP and pH‐insensitive DsRed in IR1631 worms fed with HT115, *unc‐51* RNAi and *pdr‐1* RNAi bacteria in the presence or absence of DTCA&ETCA. Magnification: 40× , scale bar: 25 μm. (F) Bar chart showing the GFP/DsRed ratio in IR1631 worms; error bars, S.D., *p < .05; **p < .01; ***p < .001, *n* = 20. (G) Representative images of Aβ3−42 deposits in CL2331 worms at 72 h and paralysis in CL4176 worms at 36 h post‐treatment with DTCA&ETCA. CL2331 worms: Magnification: 40× , scale bar: 25 μm; CL4176 worms: Magnification: 4× , scale bar: 250 μm. (H) Bar chart indicating the number of Aβ3‐42 deposits in the anterior area; error bars, S.D., **p < 0.01, *n* = 20. (I) Bar chart showing the percentage of non‐paralyzed worms (*n* > 60); error bars, S.D., ***p < .001, *n* = 3. (J) Bar chart displaying the slowing rate of N2 and BR5270 worms treated with or without DTCA&ETCA at the 72‐h time point; error bars, S.D., ***p < .001, *n* = 10. Aβ3‐42 deposits are indicated by yellow arrows; non‐paralyzed worms are indicated by red arrows. (K) Representative images of paralysis in CL4176 worms fed with HT115, *unc‐51* RNAi, *pdr‐1* RNAi, *bec‐1* RNAi and *vps‐34* RNAi bacteria in the presence or absence of DTCA&ETCA for 36 h. Magnification: 4× , scale bar: 250 μm. Non‐paralyzed worms are indicated by red arrows. (L) Bar chart showing the percentage of non‐paralyzed worms (*n* > 60); error bars, S.D., ***p < .001, *n* = 3. Results were pooled from three independent experiments.

**FIGURE 4:**
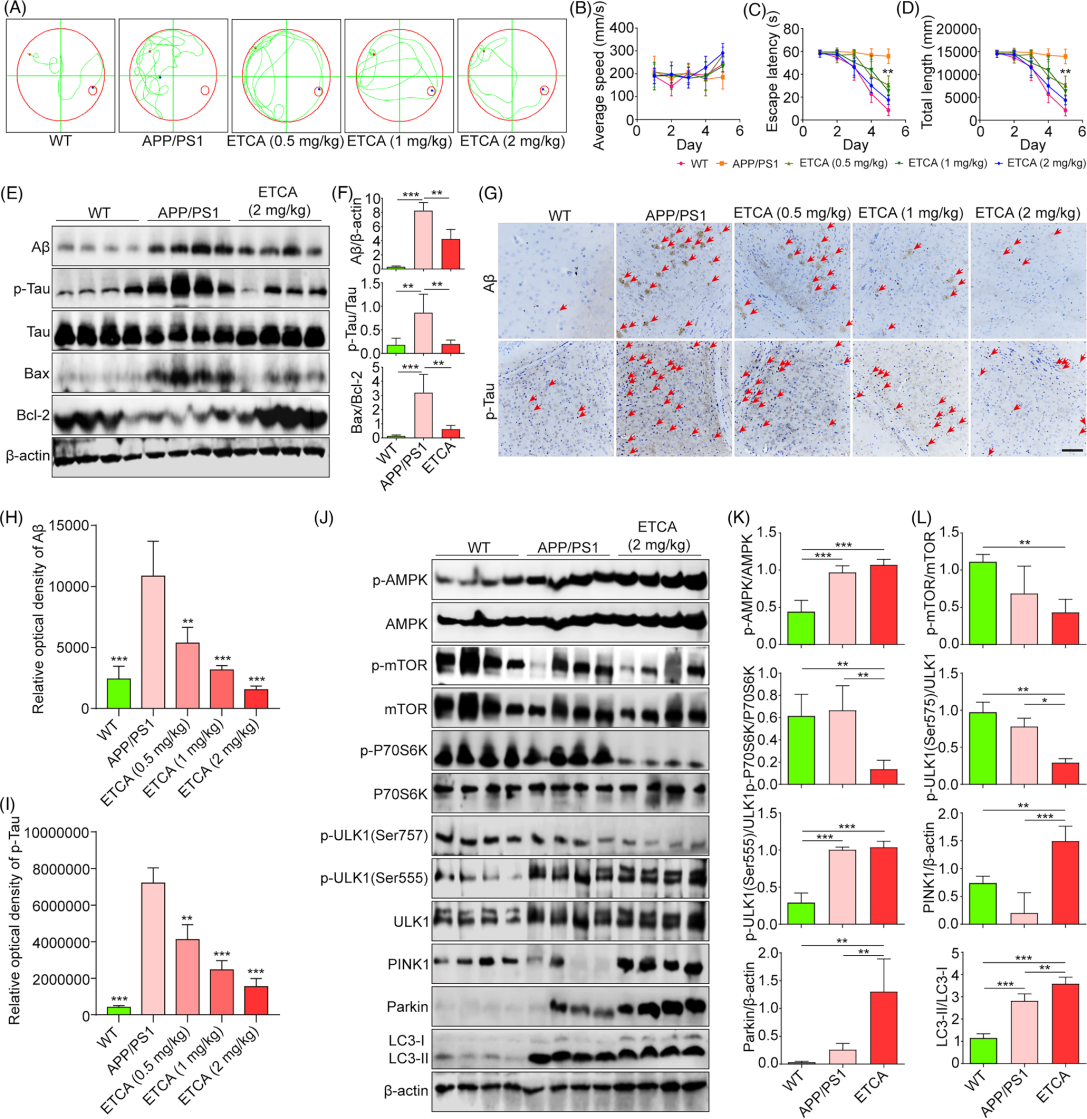
Epitrillenoside CA (ETCA) mitigates cognitive impairment and AD‐associated pathology in APP/PS1 mice via mitophagy induction. (A) Computer‐generated superimposed swimming trajectories of wide‐type and APP/PS1 mice treated with or without ETCA. (B–D) Average swimming speed, escape latency and total swimming length of wide‐type and APP/PS1 mice treated with or without ETCA; error bars, S.D., **p < .01, *n* = 8. (E) Western blotting of Aβ, p‐Tau, Tau, Bax, Bcl‐2 and β‐actin in hippocampal tissue of wide‐type and APP/SP1 mice. (F) Bar charts showing Aβ/β‐actin, p‐Tau/Tau and Bax/Bcl‐2 ratios; error bars, S.D., **p < .01, ***p < .001, *n* = 4. The original Western blot images are presented in Figure S27, where the protein molecular weight markers were labelled. (G) Representative immunohistochemistry images of Aβ and p‐Tau in hippocampal sections of wide‐type and APP/PS1 mice. Magnification: 10×, scale bar: 100 μm. Red arrows denote the presence of Aβ and p‐Tau. (H and I) Bar charts indicating the relative optical density of Aβ and p‐Tau; error bars, S.D., **p < .01; ***p < .001, *n* = 3. (J) Western blotting of p‐AMPK, AMPK, p‐P70S6K, P70S6K, p‐mTOR, mTOR, p‐ULK1(Ser555), p‐ULK1(Ser757), ULK1, PINK1, Parkin, LC3 and β‐actin. (K and L) Bar charts indicating p‐AMPK/AMPK, p‐P70S6K/P70S6K, p‐mTOR/mTOR, p‐ULK1(Ser757)/ULK1, p‐ULK1(Ser555)/ULK1, PINK1/β‐actin, Parkin/β‐actin and LC3‐II/LC3‐I ratios; error bars, S.D., *p < .05, **p < .01, ***p < .001, *n* = 4. The original Western blot images are presented in Figure S28, where the protein molecular weight markers were labelled.

In summary, DTCA&ETCA demonstrate potential as mitophagy enhancers, clearing AD‐related proteins through mTOR, AMPK/ULK1 and PINK1/Parkin signaling pathways (Figure S18). These findings enhance our understanding of DTCA&ETCA's therapeutic potential in AD treatment and validate their future clinical application. Additionally, to provide a comprehensive assessment, we intend to investigate the impact of DTCA and ETCA on toxic forms of Aβ and p‐tau oligomers in future experiments.

## CONFLICT OF INTEREST STATEMENT

The authors declare that the research was conducted in the absence of any commercial or financial relationships that could be construed as a potential conflict of interest.

## Supporting information

Supporting Information, Additional supporting information can be found online in the Supporting Information section at the end of this article.

## Data Availability

All data generated or analyzed during this study are included in this published article and its supplementary information files.
