# Supplementary material for: Two 18‐norspirostane steroidal saponins as novel mitophagy enhancers improve Alzheimer's disease
Source: Clin Transl Med. 2023 Sep 1;13(9):e1390. doi: 10.1002/ctm2.1390 (PMC10474309; doi:10.1002/ctm2.1390)
Supplement: Supplementary file 1 — Supporting Information, Additional supporting information can be found online in the Supporting Information section at the end of this article. [file CTM2-13-e1390-s001.docx]

**Supplementary material**

**Two 18-norspirostane steroidal saponins as novel mitophagy enhancers improve pathology and cognitive deficits in Alzheimer’s disease**

**Wen-Qiao Qiu^1,2†^, Lu Yu^1,†^, Chang-Long He^1,†^, Jian-Ming Wu^1^, Betty Yuen-Kwan Law^3^, Chong-Lin Yu^1^, Da-Lian Qin^1,^*, Xiao-Gang Zhou^1,^* and An-Guo Wu^1,^***

^1^*Sichuan Key Medical Laboratory of New Drug Discovery and Druggability Evaluation, Luzhou Key Laboratory of Activity Screening and Druggability Evaluation for Chinese Materia Medica, School of Pharmacy; Education Ministry Key Laboratory of Medical Electrophysiology; College of Preclinical Medicine; Southwest Medical University, Luzhou 646000, China.* fanny.qiu@outlook.com (W.-Q.Q.); yulu863@swmu.edu.cn (L.Y.); 284400200@qq.com (C.-L.H.); jianmingwu@swmu.edu.cn (J.-M.W.); 8056ycl@swmu.edu.cn (C.-L.Y.); dalianqin@swmu.edu.cn (D.-L.Q.); zxg@swmu.edu.cn (X.-G.Z.); wuanguo@swmu.edu.cn (A.-G.W.).

^2^*Department of Neurosurgery Sichuan Provincial People’s Hospital, University of Electronic Science and Technology of China, Chengdu 610000, China.* fanny.qiu@outlook.com (W.-Q.Q.).

^3^*State Key Laboratory of Quality Research in Chinese Medicine, Macau University of Science and Technology, Taipa, Macau 999078, China.* yklaw@must.edu.mo (B.Y.-K.L.).

^†^These authors contributed equally to this work

*****Corresponding author(s). Tel.: +86-17769617417; fax:+86-083031361222

E-mail address(es): dalianqin@swmu.edu.cn (Da-Lian Qin), zxg@swmu.edu.cn (Xiao-Gang Zhou), wuanguo@swmu.edu.cn (An-Guo Wu).

**Methods**

**Chemicals and reagents**

DTCA and ETCA, both with HPLC-assessed purity exceeding 98%, were previously isolated and identified from TTM by our research group ^1^. Rapamycin (Rap, T1537), 3-methyladenine (3-MA, T1879), and Compound C (CC, T6146) were procured from Topscience Co., Ltd. (Shanghai, China), while Bafilomycin A1 (Baf, B101389) was obtained from Aladdin Bio-Chem Technology Co., Ltd. (Shanghai, China). Mito-Tracker Red CMXRos was acquired from the Beyotime Institute of Biotechnology (Shanghai, China). Aβ25-35 and Aβ1-42 peptides were sourced from ChinaPeptides Ltd. (Shanghai, China). pEGFP-LC3 and mRFP-GFP tandem fluorescent-tagged LC3 (tf-LC3) plasmids were kindly provided by Tamotsu Yoshimori (Osaka University, Osaka, Japan). pEGFP-N1-APP (Addgene, #69924) was a gift from Zita Balklava and Thomas Wassmer. pRK5-EGFP-Tau-P301L (Addgene, #46908) and pRK5-EGFP-Tau (Addgene, #46904) were donated by Karen Ashe. pLVX-Puro-TagRFP-GFP-fis1(101-152end) (Mito-QC, PPL02027-4a), and pEGFP-N1 (6085-1) were purchased from the Public Protein/Plasmid Library (PPL, Nanjing, China). Antibodies targeting Bax (#14796), Bcl-2 (#3498), PI3K (#4249), p-PI3K (Tyr458, #4228), Akt (#4691), p-Akt (Ser473, #4060), mTOR (#2972), p-mTOR (Ser2448, #2971), AMPK (#9158), p-AMPK (Ser485, #2535), P70S6K (#2708), p-P70S6K (Ser371, #5536), p-ULK1 (Ser555, #5869), and p-ULK1 (Ser757, #6888) were sourced from Cell Signaling Technologies Inc. (CST, Beverly, MA, USA). Caspase-3 (#011), Caspase-9 (#0039), and LC3B (PM036) were obtained from Medical & Biological Laboratories Co., Ltd. (MBL, NGO, Japan). PARP-1 (sc-56198), GFP (sc-390394), ULK1 (sc-390904), Parkin (sc-32282), and β-actin (sc-47778) antibodies were procured from Santa Cruz Biotechnology, Inc. (CA, USA). PINK1 (23274-1-AP) antibody was purchased from Proteintech Group, Inc. (Wuhan, China).

**Cell culture**

Neuronal cell lines, encompassing HT-22, PC-12, and N2a, were acquired from the American Type Culture Collection (ATCC, Rockville, MD, USA). Wild type (WT) and Atg7-deficient mouse embryonic fibroblast cells (MEFs) were kindly provided by Masaaki Komatsu (Juntendo University, School of Medicine, Tokyo, Japan). All cells were maintained in DMEM supplemented with 10% fetal bovine serum (FBS) (Gibco, Rockville, MD, USA), 50 U/mL penicillin, and 50 μg/mL streptomycin (Invitrogen, Scotland, UK) within a 5% CO_2_ incubator at 37 °C.

**Preparations of Aβ25-35 and Aβ1-42**

Aβ25-35 and Aβ1-42 were prepared according to the previously established protocol^2^. Briefly, 1 mg of either Aβ25-35 or Aβ1-42 peptide was dissolved in a hexafluoroisopropanol (HFIP, Sigma) solution and aliquoted into 1.5 mL tubes. The HFIP solution in the tubes was evaporated under a nitrogen gas stream, resulting in a peptide film. This peptide film was then stored at -80 °C until required for further experimentation. Prior to in vitro assays, the peptide film was reconstituted in DMSO and subsequently diluted with sterilized PBS or culture medium to achieve the desired working concentrations. The solution was then incubated at 37 °C for 8 days.

**MTT** **assay**

Cells were plated in 96-well plates at a density of 5 × 10^3^ cells per well. Following treatment, the medium was replaced with fresh medium containing 0.5 mg/mL thiazolyl blue tetrazolium bromide (MTT, Sigma, St. Louis, MO, USA), and the plates were incubated at 37 °C for 4 hours. Subsequently, the medium was removed, and 150 μL of DMSO solution was added to each well. The optical density (OD) of the solutions was measured at 570 nm using a Cytation 3 microplate reader (BioTek, VT Lab, USA). Cell viability was determined using the following equation: cell viability (%) = (OD value of drug treatment / OD value of DMSO control) × 100. The reported data represent the average of three independent experiments.

**Cell transfection**

PC-12, HT-22, or N2a cells were plated in 6-well plates for 24 hours. Upon reaching a cell density of approximately 60-80%, plasmids such as tf-LC3, pEGFP-LC3, pRK5-EGFP-Tau-P301L, pRK5-EGFP-Tau, pEGFP-N1-APP, and pEGFP-N1 were introduced into the cells using ExFect2000 Transfection Reagent (Vazyme Biotech Co., Ltd., Nanjing, Jiangsu, China) following the manufacturer's guidelines. After a 24-hour transfection period, cells were exposed to the specified compounds for an additional 24 hours. Subsequently, cells were harvested for subsequent analyses, including Western blot, flow cytometry, and fluorescence imaging observation and capture.

**Western blot**

Total protein content from cells or brain tissues was extracted on ice using 1× RIPA lysis buffer (CST, Inc., Danvers, MA, USA) supplemented with protease inhibitors. Lysates were collected and centrifuged at 12,000 rpm for 10 minutes at 4 °C. Subsequently, the supernatant was transferred to 1.5 mL tubes. Protein concentrations were determined utilizing the Quick Start™ Bradford 1 × Dye Reagent (Bio-Rad, CA, USA). Equal amounts of proteins were subjected to sodium dodecyl sulfate/polyacrylamide gel (SDS-PAGE) electrophoresis and transferred onto polyvinylidene fluoride (PVDF) membranes (PALL, NYC, USA) under a 250-mA current for 1.5-2 hours. The membranes were then blocked in PBST containing 5% non-fat milk and incubated with primary antibodies (1:1000) at 4 °C overnight, followed by incubation with HRP-conjugated secondary antibodies (1:2000) at room temperature for 1 hour. After three washes with PBST, bands were visualized using the UltraSignal™ ECL Western blotting detection reagent (4A Biotech Co., Ltd., Beijing, China) and imaged with a ChemiDoc MP Imaging System (Bio-Rad, USA). Protein band intensities, representing relative expression levels, were measured using ImageJ software (National Institutes of Health, Bethesda, MD, USA). Blots shown are representative of at least three independent experiments.

**Flow cytometry analysis**

Cell viability and apoptosis: The cell viability and apoptosis were determined via flow cytometry analysis using the Annexin V-FITC/PI apoptosis detection kit (4A Biotech Co., Ltd, Beijing, China). Briefly, following treatment, cells were collected and centrifuged at 2000 rpm for 5 minutes. The supernatant was removed, and the cell pellet was resuspended in 500 μL of 1× Annexin V solution containing 4 μL of propidium iodide (PI) and 2 μL of FITC solution, following the manufacturer's guidelines. After a 15-minute incubation in the dark at room temperature, cells were analyzed using a FACSVerse flow cytometer (BD Biosciences, San Jose, CA, USA). Data acquisition and cell viability analysis were conducted using BD FACSuite v1.0.6 software (BD Biosciences, San Jose, CA, USA). Three independent experiments were performed.

Fluorescence intensity quantification: Expression levels of APP, Tau, and Tau-P301L and the cell death rate in pRK5-EGFP-Tau-P301L, pRK5-EGFP-Tau, pEGFP-N1-APP, and pEGFP-N1 transiently transfected cells were measured by determining the percentages of cells with GFP and PI signals, respectively. In brief, after transfection and treatment, cells were centrifuged at 2000 rpm for 5 minutes. The supernatant was removed, and the cell pellet was resuspended in 300 μL of PBS containing 4 μL of PI solution and incubated at room temperature for 15 minutes. Following incubation, the GFP intensity representing APP, Tau, or Tau-P301L expression was detected using the FITC channel, while cell death was detected via the PE channel. Data acquisition and analysis were performed using BD FACSuite v1.0.6 software (BD Biosciences, San Jose, CA, USA). The fluorescence of 10,000 cells was collected for each of three independent experiments.

**Fluorescence microscopy analysis**

Cells cultured on coverslips in 6-well plates were transfected with tf-LC3, pEGFP-LC3, pRK5-EGFP-Tau-P301L, pRK5-EGFP-Tau, or pEGFP-N1-APP plasmids for 24 hours. Following treatment, the medium was removed, and cells were fixed with 4% paraformaldehyde (PFA) for 15 minutes at room temperature. Subsequently, cells were washed with PBS three times and stained with DAPI solution (1:100, Beyotime, Shanghai, China) for 15 minutes. The coverslips were then air-dried and mounted using FluorSaveTM mounting media (Calbiochem, San Diego, CA, USA) for further fluorescence microscopy analysis. Representative images from five random fields were captured using a fluorescence microscope (Nikon, Japan), and fluorescence intensity was quantified with ImageJ software (National Institutes of Health, Bethesda, MD, USA). Graph presents the mean fluorescence intensity from three independent experiments

**Thioflavin-T (ThT) Fluorescence Assay**

The direct inhibitory effect of DTCA or ETCA on Aβ fibril formation was assessed by measuring fluorescence levels using the ThT reagent, following a previously described method^3^. Briefly, 20 µL of Aβ1-42 or Aβ25-35 was co-incubated with 80 µL of PBS, with or without DTCA or ETCA, at 37 °C in the dark. At 24, 48, and 72-hour time points, 10 µL aliquots of these solutions were combined with 190 µL of ThT solution (20 µM) in a black 96-well plate, followed by a 1-hour incubation at 37 °C in the dark. Subsequently, the fluorescence intensity indicative of Aβ fibril formation was quantified using a Cytation 3 microplate reader with excitation and emission wavelengths set at 450 nm and 490 nm, respectively. The background fluorescence of the control solution, containing PBS and 0.02% DMSO, was subtracted from the measured fluorescence in the Aβ-containing solution. Data represents the average of three independent experiments performed in triplicate.

**Biolayer Interferometry (BLI) Analysis**

The direct interaction of Aβ fibrils with DTCA or ETCA was assessed using biolayer interferometry (BLI) analysis, following a previously established method^3^. Briefly, Aβ fibrils were generated by incubating 200 µL of Aβ1-42 solution (50 µM) at 37 °C for 5 days. EZ-Link NHS-LC-LC-Biotin (Thermo Scientific, United States) was prepared in DMSO at a final concentration of 10 mM. Aβ fibrils were biotinylated using a 3:1 molar ratio of biotin reagent and incubated at room temperature for 1 h. Subsequently, the biotinylated Aβ1-42 solution, filtered through a Zeba desalting spin column, was added to a 96-well plate (Greiner BioOne, PN:656206). Biotinylation was confirmed by loading the mixture onto streptavidin (SA) capacity tips (ForteìBIO, Menlo Park, CA, USA) and detected using the ForteìBIO Octet Red 96e instrument (ForteìBIO, Menlo Park, CA, USA) according to the manufacturer's guidelines. Moreover, SA biosensors were pre-wetted with PBS containing 5% DMSO and 0.02% Tween for baseline recording. The biotinylated Aβ1-42 solution was immobilized onto SA tips and equilibrated in PBS with 5% DMSO and 0.02% Tween. DTCA or ETCA (6.25, 12.5, 25, 50, 100, and 200 µM) was added to the wells in 200 µL volumes, with a control solution containing an equal amount of DMSO also added. Each experiment consisted of repeated cycles involving four main steps: baseline (120 s), loading (180 s), association (120 s), and dissociation (120 s). Association and dissociation curves, as well as kinetic constants, were analyzed and acquired using ForteìBIO data analysis software (ForteìBIO, Menlo Park, CA, USA).

**Transmission electron microscopy**

HT-22 cells were exposed to DTCA, ETCA, or UA for 24 h, after which the cells were harvested and sectioned into small specimens (one dimension < 1 mm) before being fixed in Trump's fixative (Electron Microscopy Sciences) for 2 h at room temperature. Subsequently, the samples were rinsed with 0.1 M cacodylate buffer and post-fixed with 1% osmium tetroxide, followed by dehydration in ethanol and embedding using the EMbed 812 kit (Electron Microscopy Sciences). Images were acquired utilizing a transmission electron microscope (JEM-1400FLASH). Autophagic vacuole (AV)-like organelles were identified by double-membrane structures enclosing mitochondria (mt) or other organelles or vesicles, while mt were characterized by a closed membrane sac structure composed of two layers of unit membrane ^4^.

***C. elegans* strains and maintenance conditions**

All nematode strains employed in this research were procured from the Caenorhabditis Genetics Center (CGC), except when indicated otherwise. These strains were maintained on NGM plates, nourished with Escherichia coli OP50, and incubated at 20 °C. The strains utilized in the study comprised N2 (WT); DA2123, adIs 2122[lgg-1p::GFP::lgg-1+rol-6(su1006)]; BC12921, sIs10729 [rCes T12G3.1::GFP + pCeh361]; CL2331, dvIs37 [myo-3p::GFP::A-Beta (3-42) + rol-6(su1006)]; CL4176, dvIs27 [myo-3p::A-Beta (1-42)::let-851 3'UTR) + rol-6(su1006)] X; and BR5270, byIs161 [rab-3p::F3(delta)K280 + myo-2p::mCherry].

**Determination of autophagy in C. elegans**

In this investigation, the BC12921 strain expressing the p62/SQST-1-GFP fusion protein and the DA2123 strain expressing the microtubule-associated protein 1 light chain 3 beta (LC3B) autophagosome protein LGG-1 fused with GFP were employed to examine the autophagic activity of DTCA and ETCA in vivo. Briefly, strains were maintained at 20 ℃ and exposed to DTCA, ETCA, or Rap for 48 hours, starting one-day post-egg hatching. Subsequently, the nematodes were placed on a glass slide containing 0.1% NAN3 and subjected to fluorescence microscopic capture and observation using a Leica DM6B fluorescence microscope (Leica Microsystems GmbH, Germany). The fluorescence intensity, signifying p62 expression in the BC12921 strain or representing the total quantity of GFP::LGG-1/Atg8 puncta within a uniform area in the DA2123 strain, was quantified utilizing ImageJ software, as previously outlined ^5^. Graph presents the mean fluorescence intensity from three independent experiments

**Measurement of Aβ3–42 aggregation in the CL2331 strain**

An examination of Aβ3-42 aggregation was conducted using the CL2331 strain, which temperature-sensitively expresses human Aβ3-42 fused with GFP in the body wall muscle. Briefly, the nematodes were exposed to DTCA or ETCA and cultured at 23 ℃ to induce Aβ3-42 aggregation until the second day of adulthood. Following treatment, worms were placed on a glass slide containing 0.1% NaN3, and representative images were captured using a Leica DM6B fluorescence microscope (Leica Microsystems GmbH, Germany). The accumulation of amyloid deposits in the anterior region of uniform size in the CL2331 worms was assessed. Graph presents the mean fluorescence intensity from three independent experiments

**Measurement of mitophagy in the IR1631 strain**

The in vivo mitophagy effects of DTCA or ETCA were assessed in the IR1631 strain (kindly provided by Dr. Tavernarakis). Briefly, eggs from IR1631 worms grown on OP50 medium were obtained through bleaching, washed three times in M9 buffer, and allowed to hatch for 18 h. Synchronized L1 worms were then transferred to NGM plates and fed with either control HT115 bacteria or RNAi bacteria expressing double-stranded RNA of autophagy/mitophagy-related genes, such as unc-51 and pdr-1. Concurrently, DTCA or ETCA was incorporated into the NGM plates. Following treatment, worms were placed on a glass slide containing 0.1% NAN3 and examined using a Leica DM6B fluorescence microscope (Leica Microsystems GmbH, Germany) for fluorescence microscopic capture and observation. The induction of mitophagy by DTCA or ETCA was determined by calculating the fluorescence ratio of pH-sensitive GFP to pH-insensitive DsRed utilizing ImageJ software, as previously reported^6^. Graph presents the mean fluorescence intensity from three independent experiments.

**Paralysis assay in the CL4176 strain**

A paralysis assay was executed in the Caenorhabditis elegans strain CL4176, adhering to the methodology delineated in the literature^5^. Briefly, synchronized L1-stage CL4176 nematodes (100-150 per experimental condition) were placed onto NGM plates supplemented with either DTCA or ETCA, followed by a 36-hour incubation period at 16℃. Subsequently, the temperature was raised to 25℃ to initiate the expression of Aβ3-42. Paralysis evaluations were conducted at 33, 36, and 39-hour time points. Worms were considered paralyzed if they displayed bacterial "halos" around their heads, restricted head movements, or complete immobility upon gentle contact with a platinum worm pick. Each paralysis assay was performed in triplicate, with three independent trials conducted to ensure the consistency and reliability of the results.

**Food-sensing behavior assay in the BR5270 strain**

A food-sensing behavior assay was performed in BR5270 worms, as previously reported in the paper^7^. Briefly, assay plates were prepared with a ring of E. coli OP50, exhibiting an inner diameter of approximately 1 cm and an outer diameter of approximately 8 cm. Subsequent to a 48-hour treatment with either DTCA or ETCA, BR5270 nematodes were rinsed with M9 buffer and positioned at the center of a NGM agar plate, either with or without an *E. coli* OP50 lawn. After a 5-minute interval, body bends were quantified during a 20-second observation period. The reduction in body bending rate was calculated using the following formula: Slowing rate = (N _without food_ – N _with food_)/N _without food_, where N signifies the total number of body bends exhibited by the nematodes. Data represents the average of three independent experiments performed in triplicate.

**RNAi treatment in the CL4176 strain**

To elucidate whether the neuroprotective properties of DTCA and ETCA in Caenorhabditis elegans were correlated with their autophagy induction capacities, RNA interference (RNAi) bacteria were utilized to suppress the expression of crucial autophagy-associated genes in CL4176 nematodes, including unc-51, bec-1, and vps-34. Briefly, eggs from CL4176 worms cultured on OP50 medium were obtained through bleaching, subsequently washed thrice with M9 buffer, and permitted to hatch in M9 buffer for 18 hours. The synchronized L1-stage worms were transferred to NGM plates and supplied with either control HT115 bacteria or RNAi bacteria expressing double-stranded RNA targeting the three autophagy-related genes: *unc-51*, *bec-1*, and *vps-34*. Concurrently, DTCA or ETCA was incorporated into the NGM plates. Following treatment, paralysis occurrence was assessed utilizing the previously described method.

**Animals**

Amyloid precursor protein/presenilin-1 (APP/PS1) double-transgenic (Tg) mice (Jackson Laboratory, Bar Harbor, ME, USA) of C57BL/6J background expressing mutant human APP and PS1 were utilized in this study. APP/PS1 mice (model group) and WT C57BL/6J mice (control group) were maintained at 20-24℃ with 40-60% relative humidity under a 12-hour light/dark cycle, with ad libitum access to food and water. The Animal Ethics Committee of Southwest Medical University approved all animal care and experimental procedures employed (No. 20211101-003). WT C57BL/6J and 8-month-old APP/PS1 mice (equal numbers of males and females) were subjected to the Morris water maze (MWM) test for cognitive function screening. WT C57BL/6J mice in the control group received normal saline treatment. APP/PS1 mice exhibiting comparable cognitive decline were selected and randomly assigned to one of four groups: a vehicle group (normal saline treatment), a low-dose ETCA group (0.5 mg/kg ETCA treatment), a moderate-dose ETCA group (1 mg/kg ETCA treatment), or a high-dose ETCA group (2 mg/kg ETCA treatment). Mice received daily intraperitoneal (I.P.) injections at 5 p.m. with either ETCA or a blank reagent system (normal saline: PEG400 = 7:3) for 60 days. Cognitive function was assessed using the MWM test prior to sacrifice. Mice were then anesthetized with pentobarbitone sodium (30 mg/kg, I.P.) and decapitated. Brains were collected and either stored at -80 ℃ or fixed with 4% paraformaldehyde (PFA) for subsequent Western blotting and immunohistochemical analyses.

**MWM test**

All mice were subjected to the MWM test to evaluate their spatial learning capabilities and memory performance. The MWM apparatus consists of a circular, dark gray tank (120 cm diameter, 50 cm height) filled with water maintained at 24-26 ℃. An escape platform (5 cm diameter) is situated 1 cm below the water surface in the center of three quadrants. Each mouse underwent training twice daily for six consecutive days. During the trials, mice were introduced into the water from one of the four quadrants and given 60 seconds to locate the platform and remain on it for 10 seconds. Following the six-day training period, cognitive function assessment was conducted as previously described^8^. Briefly, mice were released into the water from the same quadrant, and their escape latency was recorded. Concurrently, the number of mice that entered the quadrant containing the original platform was documented using the VisuTrack Rodent Behavior Analysis System (Shanghai XinRuan Information Technology Co., Ltd., Shanghai, China).

**Immunohistochemistry**

Mouse brains fixed in 4% paraformaldehyde (PFA) were dehydrated in 95% ethanol and embedded in optimal cutting temperature (OCT) compound medium. Subsequently, frozen sections encompassing the hippocampus were prepared and mounted on glass slides. Following blockage with 5% bovine serum albumin (BSA), the sections were incubated with primary antibodies against mouse Aβ, p-Tau, LC3-II, Iba-1, NeuN, PINK1, Parkin, or GFAP (1:100 dilution) overnight at 4℃. Sections were then incubated with goat anti-rabbit secondary antibodies (1:500 dilution) at room temperature for 1 hour, followed by development using the 3,3'-diaminobenzidine (DAB) chromogen kit. Representative micrographs were obtained using an optical microscope (Nikon, Japan), and the optical density indicative of protein expression was analyzed using ImageJ software (National Institutes of Health, Bethesda, MD, USA).

**Statistical analysis**

Data in this study were derived from a minimum of three independent experiments. Values are expressed as means ± standard deviations (SDs) and were analyzed using GraphPad Prism 5.0 statistical software (San Diego, CA, USA). Group comparisons were performed using one-way analysis of variance (ANOVA) followed by the Tukey post-hoc test, with a significance level set at *p* < 0.05.

**References**

1. Wu AG, Teng JF, Wong VK, Zhou XG, Qiu WQ, Tang Y, Wu JM, et al, Novel steroidal saponin isolated from Trillium tschonoskii maxim. exhibits anti-oxidative effect via autophagy induction in cellular and Caenorhabditis elegans models. Phytomedicine, 2019, **65**:153088.

2. Qiu WQ, Pan R, Tang Y, Zhou XG, Wu JM, Yu L, Law BY, et al, Lychee seed polyphenol inhibits Abeta-induced activation of NLRP3 inflammasome via the LRP1/AMPK mediated autophagy induction. Biomed Pharmacother, 2020, **130**:110575.

3. Yu L, Wu AG, Wong VK, Qu LQ, Zhang N, Qin DL, Zeng W, et al, The New Application of UHPLC-DAD-TOF/MS in Identification of Inhibitors on beta-Amyloid Fibrillation From Scutellaria baicalensis. Front Pharmacol, 2019, **10**:194.

4. Ye X, Sun X, Starovoytov V, Cai Q, Parkin-mediated mitophagy in mutant hAPP neurons and Alzheimer's disease patient brains. Human Molecular Genetics, 2015, **24**:2938-2951.

5. Zhu Q, Qu Y, Zhou XG, Chen JN, Luo HR, Wu GS, A Dihydroflavonoid Naringin Extends the Lifespan of C. elegans and Delays the Progression of Aging-Related Diseases in PD/AD Models via DAF-16. Oxid Med Cell Longev, 2020, **2020**:6069354.

6. Palikaras K, Lionaki E, Tavernarakis N, Mitophagy Dynamics in Caenorhabditis elegans. Methods Mol Biol, 2019, **1880**:655-668.

7. Chalorak P, Dharmasaroja P, Meemon K, Downregulation of eEF1A/EFT3-4 Enhances Dopaminergic Neurodegeneration After 6-OHDA Exposure in C. elegans Model. Front Neurosci, 2020, **14**:303.

8. Xiong R, Zhou XG, Tang Y, Wu JM, Sun YS, Teng JF, Pan R, et al, Lychee seed polyphenol protects the blood-brain barrier through inhibiting Abeta(25-35)-induced NLRP3 inflammasome activation via the AMPK/mTOR/ULK1-mediated autophagy in bEnd.3 cells and APP/PS1 mice. Phytother Res, 2020.

**
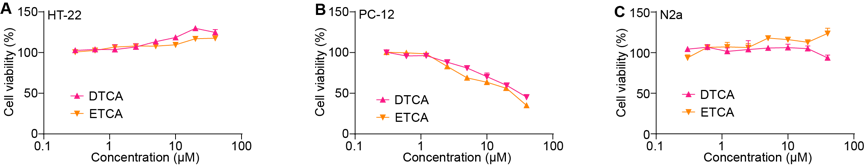
**

**Figure S1** Cytotoxicity of DTCA&ETCA in HT-22, PC-12, and N2a cells assessed via MTT assay. Line charts display the cell viability of HT-22 (A), PC-12 (B), and N2a (C) cells treated with DTCA&ETCA at specified concentrations for 24 h. n=3.


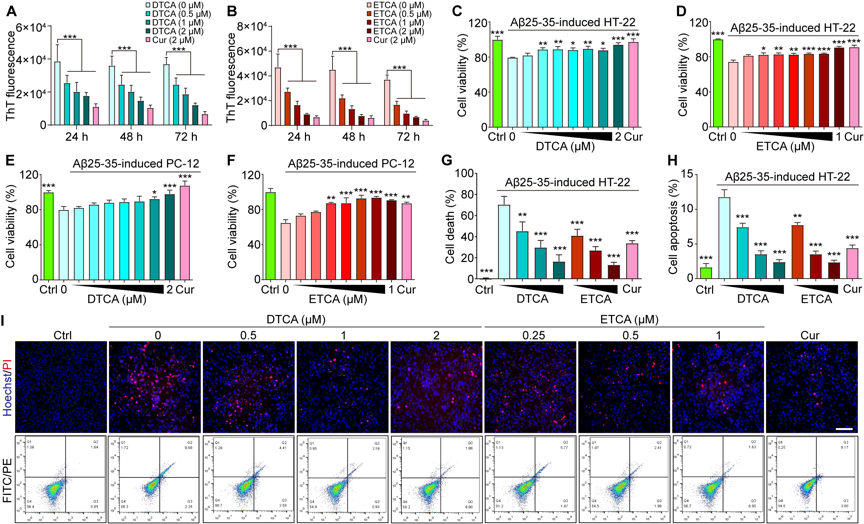


**Figure S2** DTCA&ETCA inhibit Aβ25-35 fibrillization and enhance the cell viability of Aβ25-35- or Aβ1-42-treated HT-22 cells. (A-B) Bar charts display the fluorescence intensity of solutions containing ThT reagent and Aβ25-35 with or without DTCA, ETCA, and Cur at specified concentrations; error bars, ****p* < .001, n=3. (C-F) Bar charts indicate the cell viability of Aβ25-35-treated HT-22 and PC-12 cells with or without DTCA and ETCA at specified concentrations, using Cur (10 μM) as positive control; error bars, S.D., **p* < .05; ***p* < .01; ****p* < .001, n=3. (G) The bar chart depicts the cell death of HT-22 cells determined by Hoechst/PI staining; error bars, S.D., ***p* < .01; ****p* < .001, n=3. (H) The bar chart displays the cell apoptosis of HT-22 cells determined by flow cytometry using Annexin V-PE Apoptosis Detection Kit; bars, S.D., ****p* < .001, n=3. (I) Representative images of Hoechst/PI staining and flow cytometry images of Aβ25-35-treated HT-22 cells with or without DTCA and ETCA at specified concentrations, using Cur (10 μM) as a positive control. Magnification: 20×, scale bar: 100 µm.


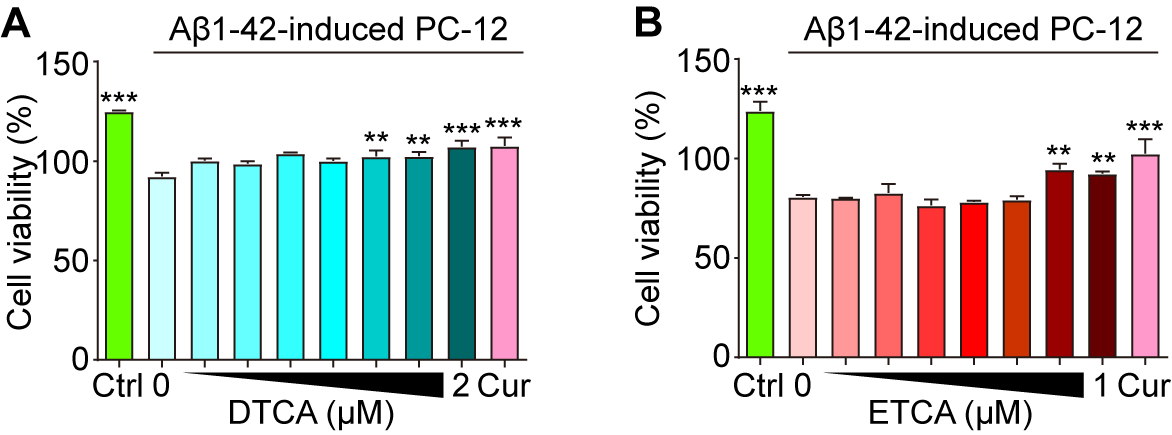


**Figure S3** Bar charts indicate the cell viability of Aβ1-42-treated PC-12 cells with or without DTCA (A) and ETCA (B) at indicated concentrations, using Cur (10 μM) as positive control; error bars, S.D., **p* < .05; ***p* < .01; ****p* < .001, n=3.


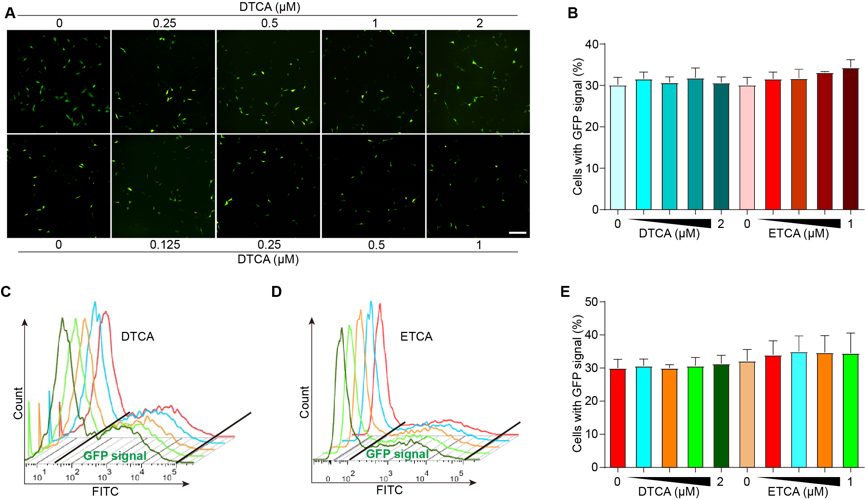


**Figure S4** Effects of DTCA&ETCA on EGFP-N1 expression in HT-22 cells. (A) Representative images of GFP signals in EGFP-N1 overexpressing HT-22 cells treated with DTCA&ETCA at specified concentrations for 24 h. Magnification: 20×, scale bar: 100 µm. (B) Bar chart indicating the percentage of HT-22 cells with GFP signals, n=3. (C, D) Representative flow cytometry images showing GFP intensity in EGFP-N1 overexpressing HT-22 cells treated with DTCA&ETCA. (E) Bar charts displaying the percentage of HT-22 cells with GFP signals; error bars, S.D, n=3.


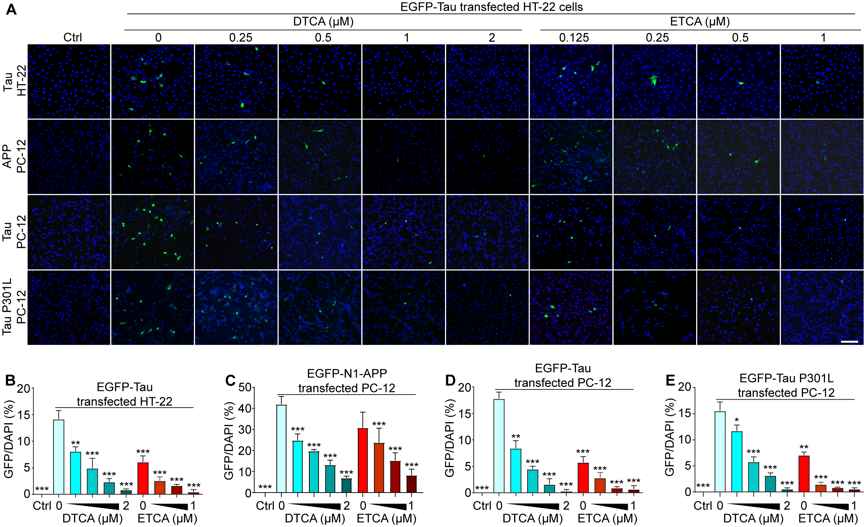


**Figure S5** DTCA&ETCA reduce EGFP-Tau expression in EGFP-Tau transfected HT-22 and EGFP-N1-APP-, EGFP-Tau-, or EGFP-Tau P301L-transfected PC-12 cells. (A) Representative images of merged EGFP-Tau and DAPI in HT-22 cells and merged EGFP-N1-APP, EGFP-Tau, or EGFP-Tau P301L and DAPI in PC-12 cells. Magnification: 20×, scale bar: 100 µm. (B-E) Bar charts indicate GFP/DAPI ratios in cells; error bars, S.D., **p* < .05, ***p* < .01, ****p* < .001, n=3.


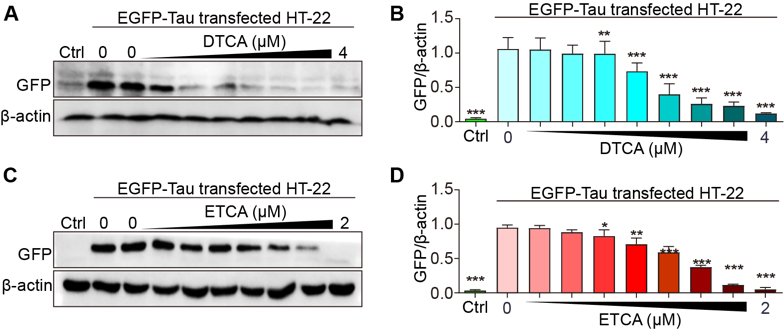


**Figure S6** DTCA&ETCA decrease GFP expression in EGFP-Tau transfected HT-22 cells. (A, C) Western blotting of GFP and β-actin in EGFP-Tau transfected HT-22 cells treated with DTCA and ETCA at indicated concentrations. The original Western blot images are presented in Figure S20, where the protein molecular weight markers were labeled. (B, D) The relative protein expression of Tau P301L is indicated by the ratio of GFP-tagged Tau P301L to β-actin using a GFP antibody, represented as GFP/β-actin. Bar charts indicate GFP/β-actin in HT-22 cells; bars, S.D., **p* < .05, ***p* < .01, ****p* < .001, n=3.


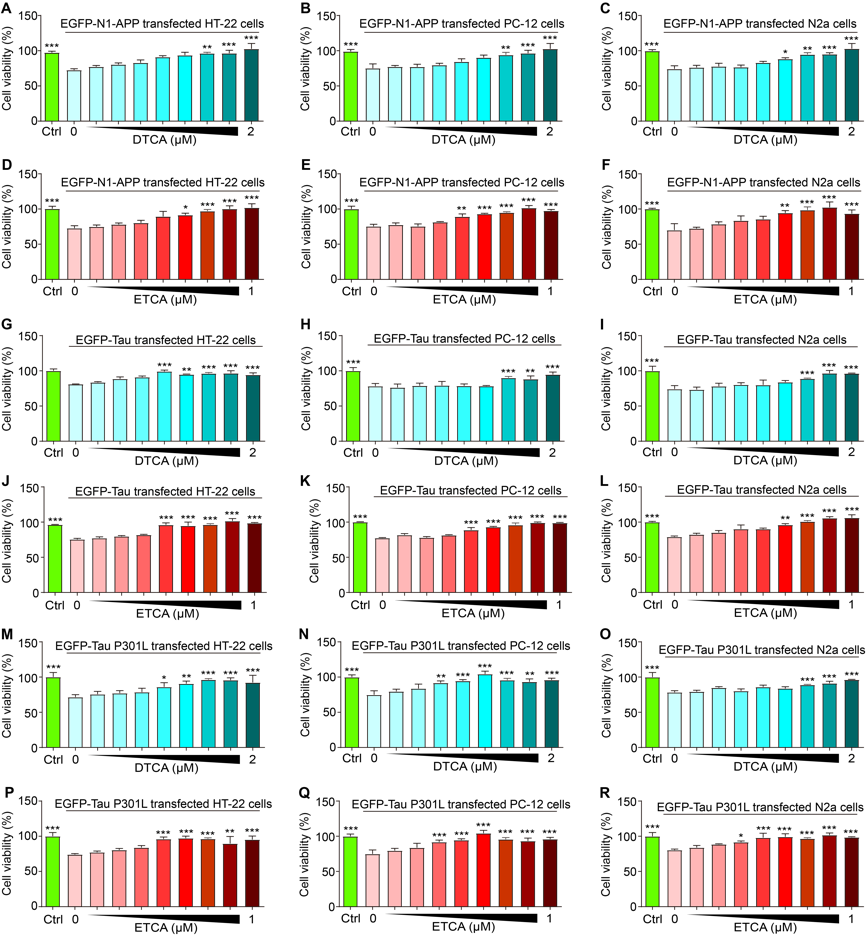


**Figure S7** DTCA&ETCA enhance the cell viability of EGFP-N1-APP transfected HT-22, PC-12, and N2a cells. (A-C) Bar charts representing cell viability in EGFP-N1-APP transfected HT-22, PC-12, and N2a cells treated with DTCA at specified concentrations. (D-F) Bar charts showing cell viability in EGFP-N1-APP transfected HT-22, PC-12, and N2a cells treated with ETCA at indicated concentrations; error bars, S.D., **p* < .05; ***p* < .01; ****p* < .001, n=3. (G-I) Bar charts displaying cell viability in EGFP-Tau overexpressing HT-22, PC-12, and N2a cells treated with DTCA at indicated concentrations; error bars, S.D., ***p* < .01, ****p* < .001, n=3. (J-L) Bar charts showing cell viability in EGFP-Tau overexpressing HT-22, PC-12, and N2a cells treated with ETCA at indicated concentrations; error bars, S.D., ***p* < .01, ****p* < .001, n=3. (M-O) Bar charts showing the cell viability in EGFP-Tau P301L overexpressing HT-22, PC-12, and N2a cells treated with DTCA at indicated concentrations; error bars, S.D., **p* < .05, ***p* < .01, ****p* < .001, n=3. (P-R) Bar charts showing cell viability in EGFP-Tau P301L overexpressing HT-22, PC-12, and N2a cells treated with ETCA at indicated concentrations; bars, S.D., **p* < .05, ***p* < .01, ****p* < .001, n=3.


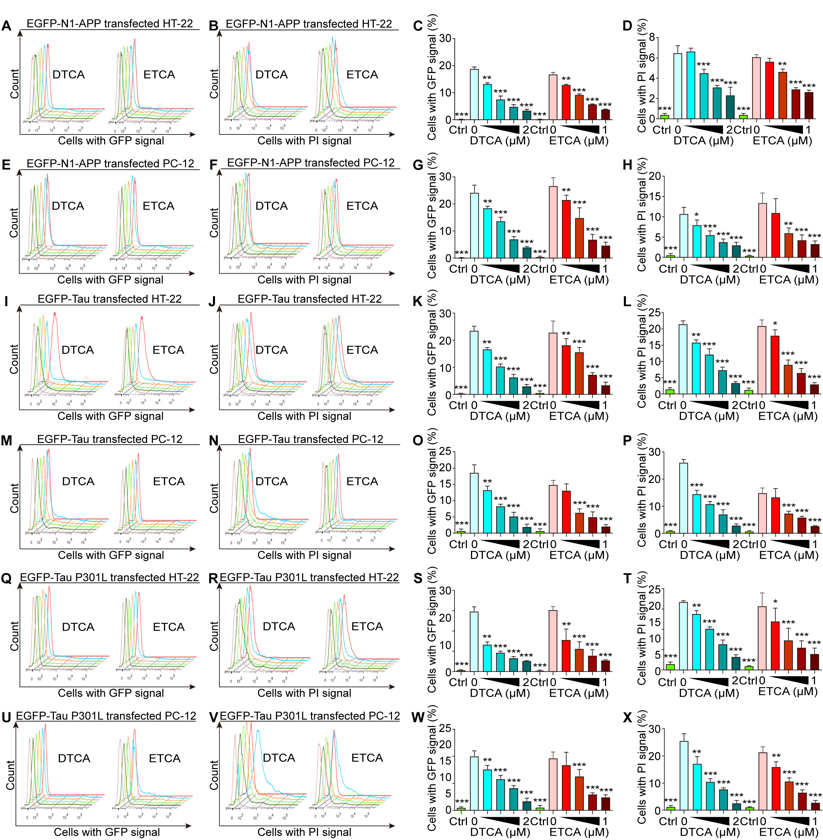


**Figure S8** DTCA&ETCA attenuate GFP intensity and cell death in EGFP-N1-APP-, EGFP-Tau- or EGFP-Tau P301L-transfected HT-22 and PC-12 cells. (A, B, E, F) Representative flow cytometry images displaying GFP and PI signal intensities in EGFP-N1-APP overexpressing HT-22 and PC-12 cells stained with PI and treated with DTCA&ETCA. (C, G) Bar charts indicate the percentage of GFP-positive HT-22 and PC-12 cells; error bars, S.D., ***p* < .01, ****p* < .001, n=3. (D, H) Bar charts indicate the percentage of PI-positive HT-22 and PC-12 cells; error bars, S.D., **p* < .05, ***p* < .01, ****p* < .001, n=3. (I, J, M, N) Representative flow cytometry images displaying GFP and PI signal intensities in EGFP-Tau overexpressing HT-22 and PC-12 cells stained with PI and treated with DTCA&ETCA. (K, O) Bar charts indicate the percentage of GFP-positive HT-22 and PC-12 cells; error bars, S.D., ***p* < .01, ****p* < .001, n=3. (L, P) Bar charts indicate the percentage of PI-positive HT-22 and PC-12 cells; error bars, S.D., **p* < .05, ***p* < .01, ****p* < .001, n=3. (Q, R, U, V) Representative flow cytometry images of GFP and PI signal intensities in EGFP-Tau P301L overexpressing HT-22 and PC-12 cells stained with PI and treated with DTCA&ETCA. (S, W) Bar charts indicate the percentage of GFP-positive HT-22 cells; error bars, S.D., ***p* < .01, ****p* < .001, n=3. (T, X) Bar charts indicate the percentage of PI-positive HT-22 cells; error bars, S.D., **p* < .05, ***p* < .01, ****p* < .001, n=3.


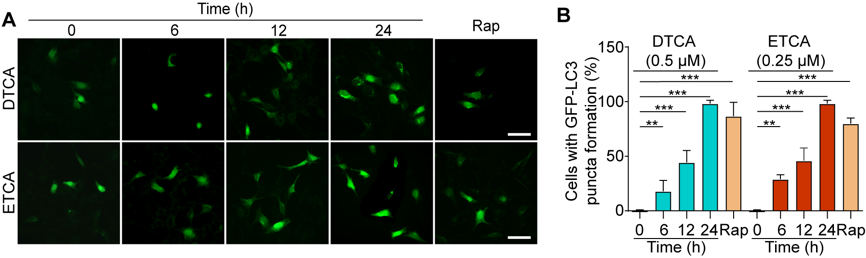


**Figure S9** DTCA&ETCA increase the percentage of cells with GFP-LC3 puncta formation in HT-22 cells. Representative GFP-LC3 puncta images in HT-22 cells treated with DTCA (0.5 μM), ETCA (0.25 μM), and Rap (10 μM) at indicated time points. Magnification: 40×, scale bar: 100 µm. (B) The bar chart shows the parentage of cells with GFP-LC3 puncta formation in HT-22 cells; error bars, S.D., ***p* < .01; ****p* < .001, n=3.


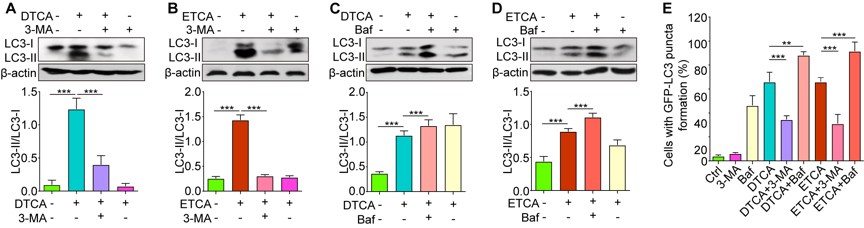


**Figure S10** 3-MA and Baf inhibit the ratio of LC3-II/LC3-I and the percentage of cells with GFP-LC3 puncta formation in DTCA- or ETCA-treated HT-22 cells. (A-D) Western blot of LC3 and β-actin in HT-22 cells treated with DTCA (0.5 µM) and ETCA (0.25 µM) with or without CC (5 μM) or Baf (5 nM) for 24 h. Bar charts indicate the quantification of LC3-II/LC3-I; bars represent S.D., ****p* < .001, n=3. The original Western blot images are presented in Figure S21, where the protein molecular weight markers were labeled. (E) Quantification of the percentage of cells with GFP-LC3 puncta formation in HT-22 cells treated with DTCA (0.5 µM) and ETCA (0.25 µM) with or without CC (5 μM) or Baf (5 nM) for 24 h; bars represent S.D., ***p* < .01, ****p* < .001, n=3.


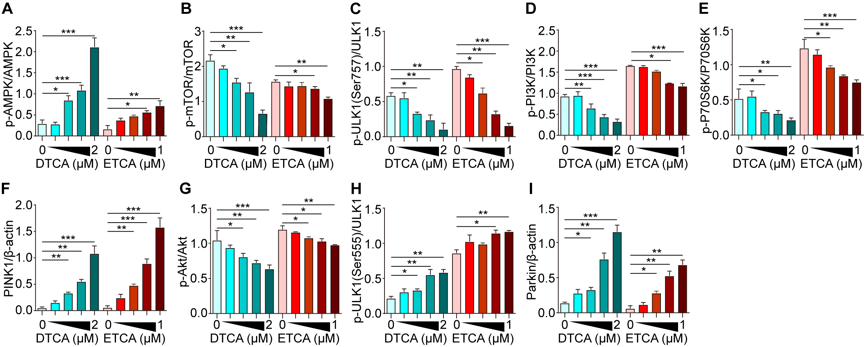


**Figure S11** Quantitative analysis of the proteins in Figure 2H; error bars, S.D., **p* < .05, ***p* < .01, ****p* < .001, n=3.


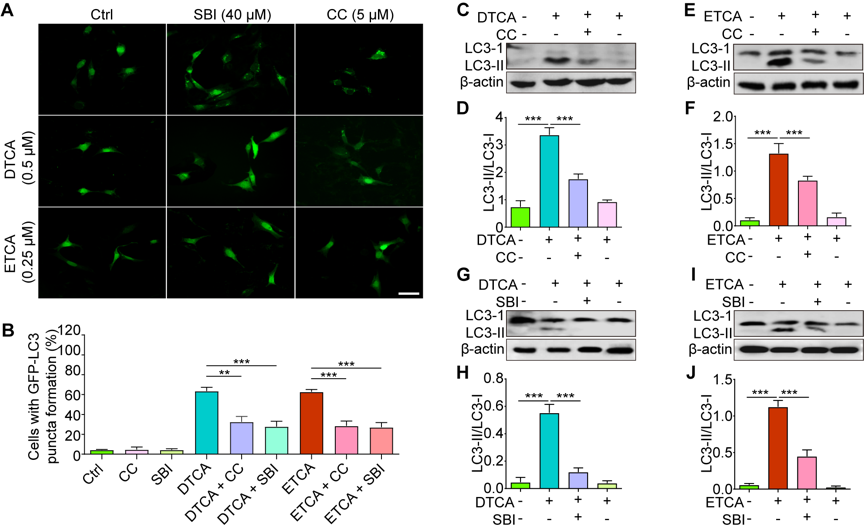


**Figure S12** SBI and CC inhibit the percentage of cells with GFP-LC3 puncta formation and the ratio of LC3-II/LC3-I in DTCA- or ETCA-treated HT-22 cells. (A) Representative GFP-LC3 puncta in HT-22 cells expressing pEGFP-LC3 and treated with DTCA and ETCA with or without CC and SBI for 24 h; Magnification: 40×, scale bar: 100 µm. (B) Percentage of cells displaying GFP-LC3 puncta; bars represent S.D., ***p* < .01; ****p* < .001, n=3. (C, E) Western blot of LC3 and β-actin in HT-22 cells treated with DTCA (0.5 µM) and ETCA (0.25 µM) with or without CC (5 μM) for 24 h. The original Western blot images are provided in Figure S23, where the protein molecular weight markers were labeled. (D, F) Quantification of LC3-II/LC3-I; bars represent S.D., ****p* < .001, n=3. (G, I) Western blot of LC3 and β-actin in HT-22 cells treated with DTCA (0.5 µM) and ETCA (0.25 µM) with or without SBI (40 μM) for 24 h. The original Western blot images are presented in Figure S23, where the protein molecular weight markers were labeled. (H, J) Quantification of LC3-II/LC3-I; bars represent S.D., ****p* < .001.


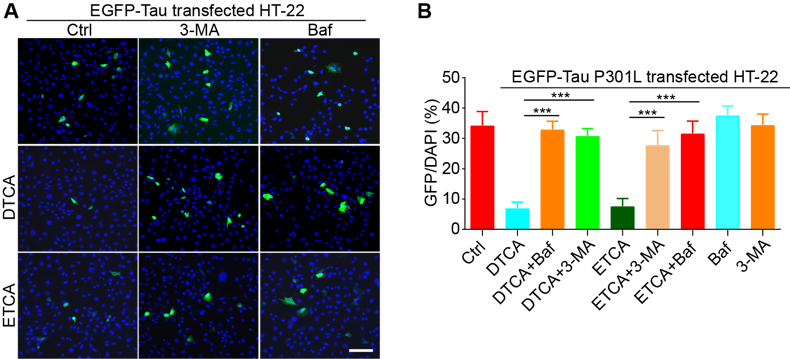


**Figure S13** 3-MA and Baf counteract DTCA and ETCA-induced decrease in EGFP-Tau expression in EGFP-Tau transfected HT-22 cells. (A) Representative images of merged EGFP-Tau and DAPI in EGFP-Tau transfected HT-22 and PC-12 cells, stained with DAPI and treated with DTCA (0.5 μM) and ETCA (0.25 μM) in the presence or absence of 3-MA (5 mM) and Baf (5 nM). Magnification: 20×, scale bar: 100 µm. (B) Bar chart showing GFP/DAPI ratios in HT-22 cells; bars, S.D., ****p* < .001, n=3.


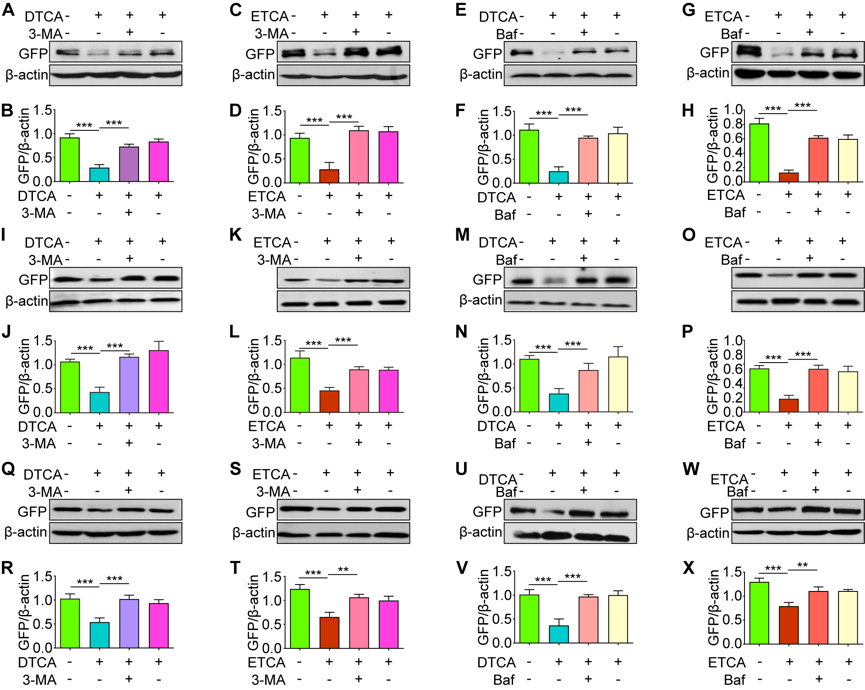


**Figure S14** 3-MA and Baf counteract DTCA and ETCA-induced decrease in GFP expression in EGFP-N1-APP-, EGFP-Tau-, or EGFP-Tau P301L-transfected HT-22 cells. (A, C, E, G) Western blotting of GFP and β-actin in EGFP-N1-APP transfected HT-22 cells treated with DTCA (0.5 μM) and ETCA (0.25 μM) in the presence or absence of 3-MA (5 mM) and Baf (5 nM). (B, D, F, H) The relative protein expression of APP is indicated by the ratio of GFP-tagged APP to β-actin using a GFP antibody, represented as GFP/β-actin. Bar charts showing GFP/β-actin ratios in EGFP-N1-APP transfected HT-22 cells; error bars, S.D., ****p* < .001, n=3. (I, K, M, O) Western blotting of GFP and β-actin in EGFP-Tau transfected HT-22 cells treated with DTCA (0.5 μM) and ETCA (0.25 μM) in the presence or absence of 3-MA (5 mM) and Baf (5 nM). (J, L, N, P) The relative protein expression of Tau is indicated by the ratio of GFP-tagged Tau to β-actin using a GFP antibody, represented as GFP/β-actin. Bar charts showing GFP/β-actin ratios in EGFP-Tau transfected HT-22 cells; error bars, S.D., ****p* < .001, n=3. (Q, S, U, W) Western blotting of GFP and β-actin in EGFP-Tau P301L transfected HT-22 cells treated with DTCA (0.5 μM) and ETCA (0.25 μM) in the presence or absence of 3-MA (5 mM) and Baf (5 nM). (R, T, V, X) The relative protein expression of Tau P301L is indicated by the ratio of GFP-tagged Tau P301L to β-actin using a GFP antibody, represented as GFP/β-actin. Bar charts showing GFP/β-actin ratios in EGFP-Tau P301L transfected HT-22 cells; error bars, S.D., ***p* < .01; ****p* < .001, n=3. The original Western blot images are presented in Figure S24, where the protein molecular weight markers were labeled.


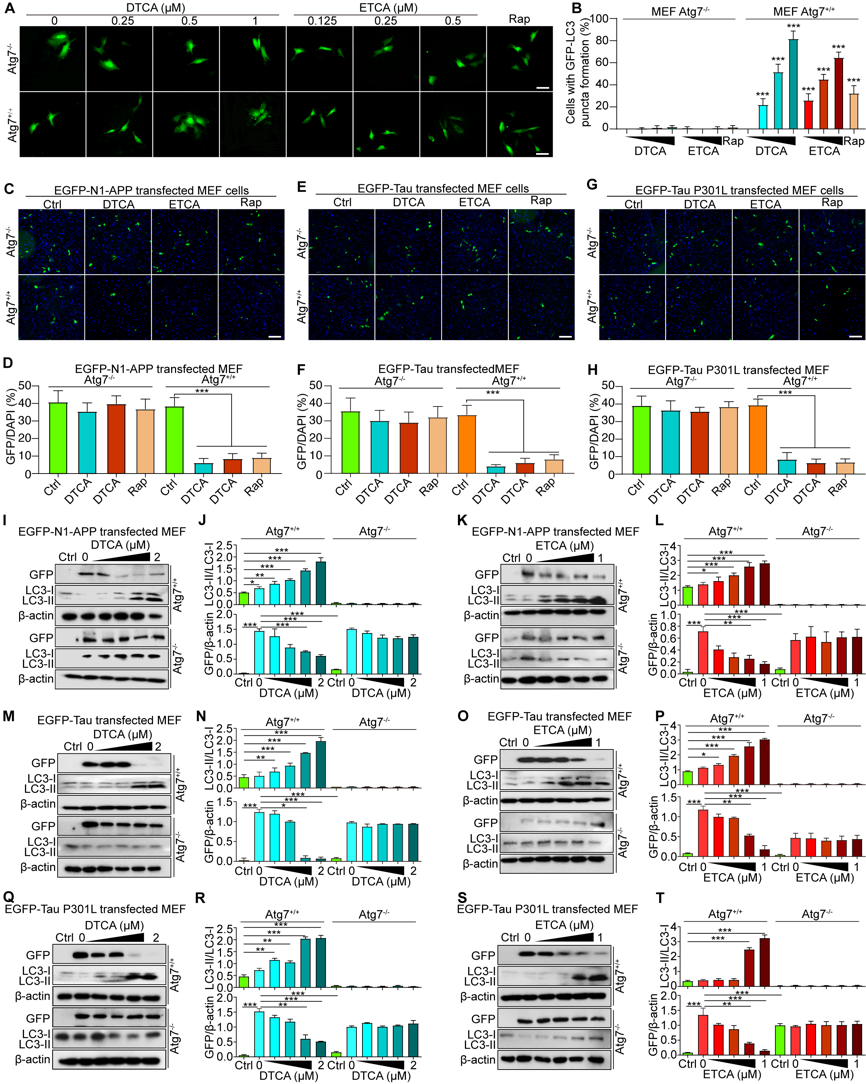


**Figure S15** DTCA&ETCA promote the autophagic degradation of AD-related proteins via Atg7 gene. (A) GFP-LC3 puncta in pEGFP-LC3-expressing wild type MEF and Atg7 deficient MEF cells treated with DTCA and ETCA for 24 h; Magnification: 40×, scale bar: 100 µm. (B) Percentage of cells with GFP-LC3 puncta; bars represent S.D., ***p < .001, n=3. (C, E, G) Representative images of EGFP-N1-APP-, EGFP-Tau-, or EGFP-Tau P301L-expressing wild-type MEF and Atg7 deficient MEF cells treated with DTCA (0.5 µM), ETCA (0.25 µM), and Rap (10 µM). Magnification: 20×, scale bar: 100 µm. (D, F, H) Percentage of cells with GFP signal relative to DAPI in EGFP-N1-APP-, EGFP-Tau-, or EGFP-Tau P301L-expressing wild-type MEF cells and Atg7 deficient MEF cells; error bars, S.D., ****p* < .001, n=3. (I, K, M, O, Q, S) Western blotting of GFP, LC3, and β-actin in EGFP-N1-APP-, EGFP-Tau-, or EGFP-Tau P301L-expressing wild-type MEF and Atg7 deficient MEF cells treated with DTCA and ETCA at indicated concentrations for 24 h. The original Western blot images are presented in Figure S25, where the protein molecular weight markers were labeled. (J, L, N, P, R, T) The relative protein expression of APP, Tau, or Tau P301L is indicated by the ratio of GFP-tagged APP, Tau, or Tau P301L to β-actin using a GFP antibody, represented as GFP/β-actin. Bar chart showing the quantification of LC3-II/LC3-I and GFP/β-actin; error bars, S.D., **p* < .05; ***p* < .01; ****p* < .001, n=3.


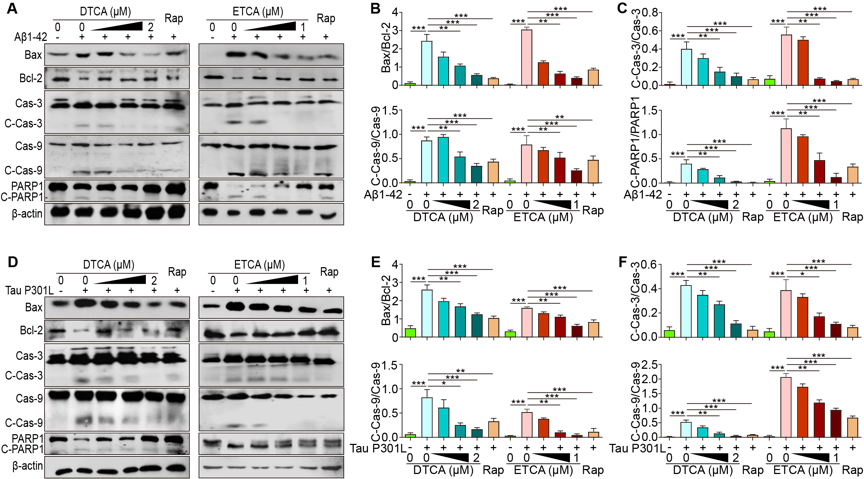


**Figure S16** DTCA&ETCA attenuate AD-associated proteins-induced apoptosis in HT-22 cells. (a) Western blotting of Bax, Bcl-2, Cas-3, C-Cas-3, Cas-9, C-Cas-9, PARP1, C-PARP1, and β-actin in Aβ1-42-treated HT-22 cells with or without DTCA, ETCA, and Rap. (b, c) Quantification of indicated protein ratios; error bars, S.D., ***p* < 0.01; ****p* < 0.001, n=3. (d) Western blotting images of Bax, Bcl-2, Cas-3, C-Cas-3, Cas-9, C-Cas-9, PARP1, C-PARP1, and β-actin in EGFP-Tau P301L-expressing HT-22 cells with or without DTCA, ETCA, and Rap. (e, f) Quantification of indicated protein ratios; error bars, S.D., **p* < .05; ***p* < .01; ****p* < .001, n=3. The original Western blot images are presented in Fig. S26, where the protein molecular weight markers were labeled.


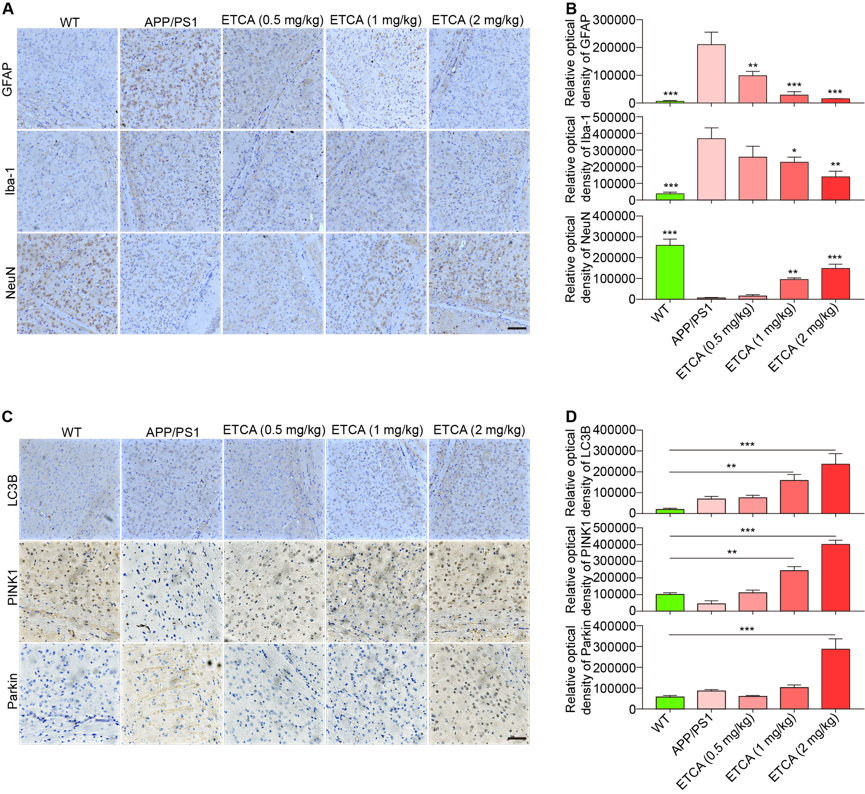


**Figure S17** ETCA suppresses inflammatory response, enhances neuronal viability, and upregulates LC3B, PINK1, and Parkin expression. (A, C) Representative immunohistochemistry images of GFAP, Iba-1, NeuN, LC3B, PINK1, and Parkin in hippocampal sections of WT and APP/PS1 mice. Magnification: 10×, scale bar: 100 μm. (C, D) Bar charts showing the relative optical density of GFAP, Iba-1, NeuN, LC3B, PINK1, and Parkin; error bars, S.D., **p* < .05, ***p* < .01, ****p* < .001, n=3.


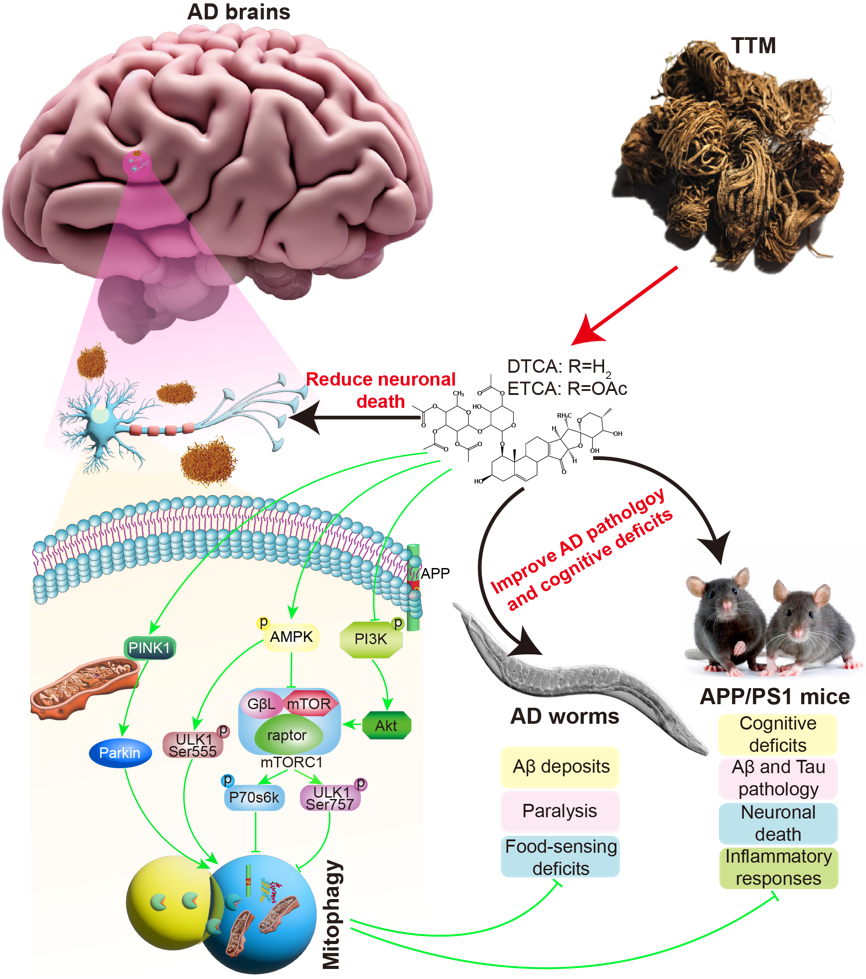


**Figure S18 A schematic diagram of the study.** Natural 18-norspirostane steroidal saponins, DTCA&ETCA, derived from TTM, impede Aβ fibrillization and cytotoxicity while facilitating the clearance of APP, Tau, and Tau-P301L in neuronal cells, *C. elegans*, and APP/PS1 mice. These effects are achieved by activating mitophagy through mTOR, AMPK/ULK1, and PINK1/Parkin signaling pathways.

**Figure S19** The original Western blotting images of Figure 1.

**Figure S20** The original Western blotting images of Figure S9.

**Figure S21** The original Western blotting images of Figure 2A, 2B, and S11.

**Figure S22** The original Western blotting images of Figure 4A.

**Figure S23** The original Western blotting images of Figure S13.

**Figure S24** The original Western blotting images of Figure S14.

**Figure S25** The original Western blotting images of Figure S15.

**Figure S26** The original Western blotting images of Figure S16.

**Figure S27** The original Western blotting images of Figure 4E.

**Figure S28** The original Western blotting images of Figure 4J.
